# Tertiary motifs as building blocks for the design of protein‐binding peptides

**DOI:** 10.1002/pro.4322

**Published:** 2022-05-10

**Authors:** Sebastian Swanson, Venkatesh Sivaraman, Gevorg Grigoryan, Amy E. Keating

**Affiliations:** ^1^ Department of Biology Massachusetts Institute of Technology Cambridge Massachusetts USA; ^2^ Department of Computer Science Dartmouth College Hanover New Hampshire USA; ^3^ Department of Biological Engineering Massachusetts Institute of Technology Cambridge Massachusetts USA; ^4^ Koch Center for Integrative Cancer Research Massachusetts Institute of Technology Cambridge Massachusetts USA

**Keywords:** computational protein design, de novo design, protein interface, peptide binder, peptide structure coverage, tertiary motif, TRAF6

## Abstract

Despite advances in protein engineering, the de novo design of small proteins or peptides that bind to a desired target remains a difficult task. Most computational methods search for binder structures in a library of candidate scaffolds, which can lead to designs with poor target complementarity and low success rates. Instead of choosing from pre‐defined scaffolds, we propose that custom peptide structures can be constructed to complement a target surface. Our method mines tertiary motifs (TERMs) from known structures to identify surface‐complementing fragments or “seeds.” We combine seeds that satisfy geometric overlap criteria to generate peptide backbones and score the backbones to identify the most likely binding structures. We found that TERM‐based seeds can describe known binding structures with high resolution: the vast majority of peptide binders from 486 peptide‐protein complexes can be covered by seeds generated from single‐chain structures. Furthermore, we demonstrate that known peptide structures can be reconstructed with high accuracy from peptide‐covering seeds. As a proof of concept, we used our method to design 100 peptide binders of TRAF6, seven of which were predicted by Rosetta to form higher‐quality interfaces than a native binder. The designed peptides interact with distinct sites on TRAF6, including the native peptide‐binding site. These results demonstrate that known peptide‐binding structures can be constructed from TERMs in single‐chain structures and suggest that TERM information can be applied to efficiently design novel target‐complementing binders.

## 1 INTRODUCTION

The ability to design custom binders of novel protein targets would greatly facilitate the development of new medicines and tools for research.^1^, ^2^ Protein‐based binders, such as antibodies or peptides, are currently obtained by directed evolution, a resource‐intensive process that can only consider a limited number of designs.^3^ Structure‐based computational design can complement experimental methods by exploring a much larger number of candidates and by providing interpretable structural models of the designed proteins.^4^ Computational methods have been used to design binders with important properties including site‐specific interaction^5^ and paralog specificity.^6^, ^7^ Despite these achievements, the vast majority of computationally designed binders do not show any measurable affinity for their target, motivating the development of new methods.

The majority of methods for constructing de novo protein backbones, such as parametric equations,^8^, ^9^ fragment assembly protocols,^10^, ^11^, ^12^ and deep‐learning hallucination methods,^13^ are geared towards designing structures that are likely to fold, but these structures may not be optimal for performing biological functions such as binding. Pioneering design efforts have repurposed natural and de novo protein structures as binders by docking them to the target protein and scoring the potential binding modes to identify top candidates.^14^, ^15^ This approach has a consistently low success rate and must be coupled with high‐throughput experimental screening to identify leads and optimize affinity.^5^, ^14^, ^16^ While it is difficult to diagnose all of the factors that contribute to the low success rates, it is likely that designed binders do not form sufficiently complementary interfaces. Indeed, analyses of designed binders have found that unsuccessful designs generally make fewer contacts with the target protein surface, compared to successful designs.^17^ In contrast, natural, high‐affinity protein–protein and peptide‐protein interfaces are consistently well‐packed,^18^, ^19^ and binders designed by expanding upon experimentally validated binding motifs exhibit higher success rates.^20^, ^21^ These observations suggest that methods for de novo binder design should prioritize structures with high interface complementarity, that is, structures able to make extensive, well‐packed interactions with the target.

Analysis of the Protein Data Bank (PDB) has shown that protein structure exhibits remarkable degeneracy at the level of small structural motifs, such as rotamers and simple backbone fragments.^22^, ^23^ Many advances in protein design have harnessed these structural motifs, motivating us to consider whether motifs could also be applied to the problem of binder design. In particular, we considered whether tertiary motifs (TERMs), which are recurring, compact structural elements that can include residues distant in the protein sequence,^24^ could be used to generate peptide structures that make favorable interactions with the target protein. Repeating instances of TERMs, in evolutionarily unrelated proteins and different contexts, effectively capture the relationship between sequence and structure. This observation is the basis of the sequence‐design method dTERMen.^25^ dTERMen has been applied to successfully design new high‐affinity BCL‐2 protein‐binding peptides and to redesign the surface of mCherry, in both cases by generating sequences compatible with a pre‐defined backbone structure.^25^, ^26^ More recently, TERMs have been used to construct new protein structures, as demonstrated by Mackenzie et al., who converted large multi‐segment TERMs into compact mini‐proteins.^27^ Previous work has applied TERMs to peptide‐protein docking,^28^, ^29^ as well as peptide design,^30^ by using them to generate candidate peptide binding poses.

Building on the success of TERM‐based approaches, in this article we consider a new strategy for designing binders of a protein target: computational assembly of peptide backbones from target‐complementing protein fragments. Our method uses TERMs that match part of the protein surface to generate target‐complementing interface seeds. Sets of overlapping seeds are joined together to create longer peptide backbones, which are then scored to assess their compatibility with the protein target. The success of this approach requires that the PDB contain appropriate structural elements that can be identified and then positioned to construct native‐like peptide‐protein interfaces. To evaluate this premise, we first tested whether TERMs from single‐chain protein structures can describe interfaces from high‐resolution structures of known peptide‐protein complexes.^31^ We analyzed seeds generated around the peptide‐binding sites of 486 proteins and found that TERM‐based seeds can cover and reconstruct known peptide backbone structures with sufficient accuracy to support the design of native‐like sequences. We present a computational proof‐of‐concept in which we used this approach to design novel peptide binders of the human protein TRAF6. The top candidate binders were predicted by Rosetta to form plausible interfaces with the known binding site as well as new sites on the target protein. Our study provides insights into similarities between structural motifs in single‐chain structures and peptide‐protein interfaces and establishes the feasibility of using TERM‐based approaches for interface design that can now be carried forward to experimental testing.

## 2 RESULTS

We first present a brief summary of our method for peptide design and then describe a series of computational benchmarks using known peptide‐protein structures. We conclude with a demonstration of our approach in an application to de novo design. All technical details are described in the Supplementary Methods.

### 2.1 A computational method for de novo peptide design

We devised a method to generate surface‐complementing peptide backbone structures from TERMs. The backbone design process proceeds in three steps. First, surface‐complementing protein fragments, which we dub interface *seeds*, are generated around the target protein using TERMs. Second, peptide backbones are generated by combining seeds that satisfy geometric overlap criteria. Finally, peptide backbones are ranked by scoring the interface formed with the target protein. Existing methods, such as dTERMen or Rosetta, can then be used to select a peptide sequence that stabilizes the peptide‐protein interaction for the highest‐ranked backbones.^25^, ^32^, ^33^

**FIGURE 1:**
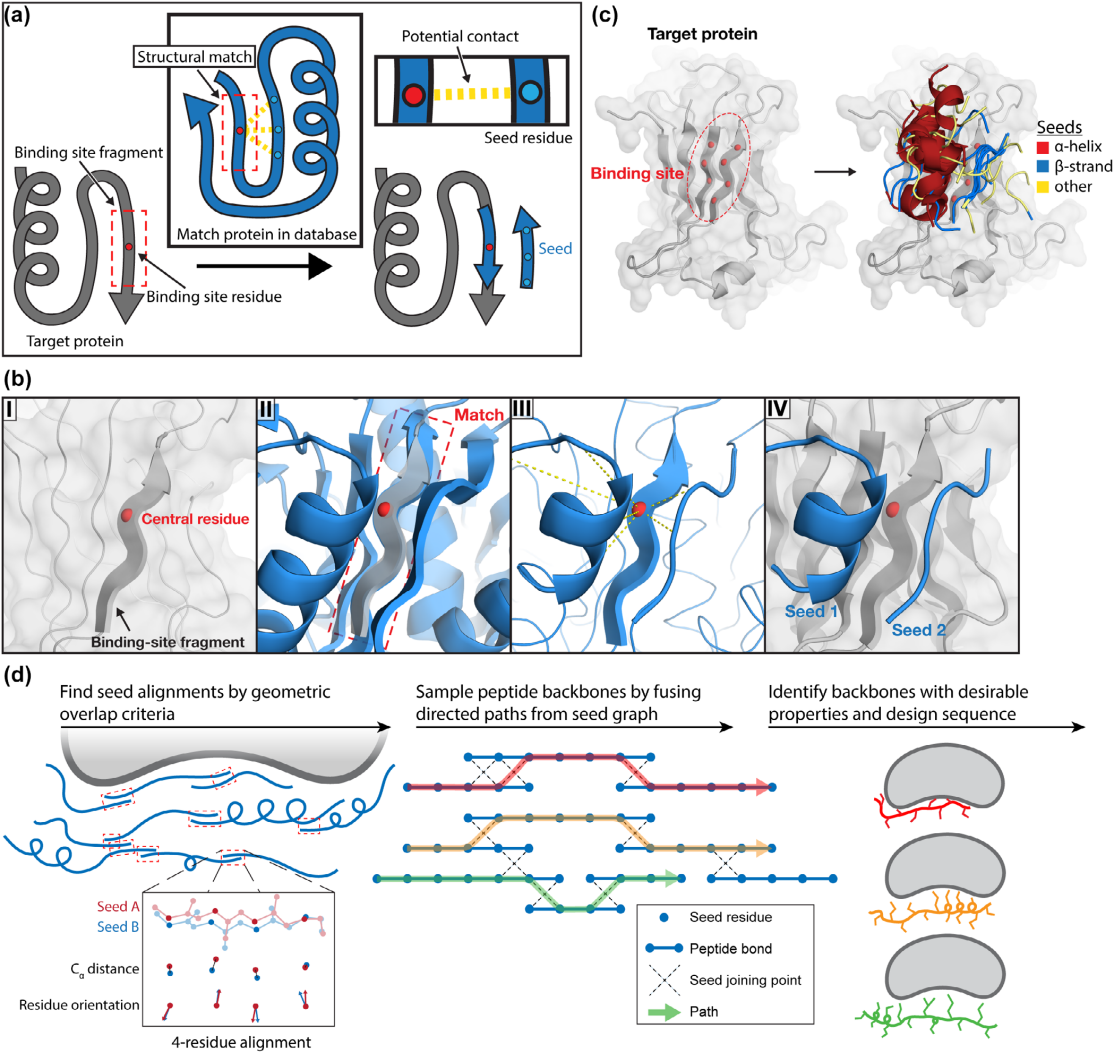
A computational method for de novo peptide design (a) Seeds are generated around a target protein by extracting related tertiary fragments from the PDB. (b) A seed is extracted from a structural match. (I) a binding‐site fragment is defined around a residue in the target protein (PDB: 1LB6), (II) a match is identified in another protein in the database, (III) seed residues with the potential to contact the central residue of the match are used to generate seeds, (IV) the seeds are placed in the target protein binding site. (c) A binding site is defined by selecting residues on the target protein and seeds are generated around it. In this example, a small sample (~30) of all seeds is shown and colored by secondary structure assigned by STRIDE. (d) Overview of the peptide design method

Seeds are extracted from known structures that contain structural motifs matching the target protein by the following procedure (Figure 1a,b). The user‐specified candidate binding site is defined by selecting target protein surface residues. A binding‐site fragment is defined around each residue by including flanking residues in the chain and, in some cases, spatially proximal residues. A database of known structures is searched for matches to the binding‐site fragment that satisfy a pre‐determined root‐mean‐square‐deviation (RMSD) cutoff over backbone atoms. When a match is identified, the structure containing the match is searched for residues with the potential to contact the central residue of the match. Each of these residues, along with its flanking residues in the chain, is placed into the context of the target binding site to create a seed. The exact pose of each seed is determined by the structural alignment. We use this protocol to rapidly generate hundreds of thousands of seeds around a potential binding site (Figure 1c).

We combined seeds to generate diverse binding modes, some with complementarity to an extensive region of the protein surface (Figure 1d). Inspired by SEWING,^34^ we developed a graph‐based path traversal algorithm for enumerating sets of seed residues that could be joined together to form a larger backbone. Seed‐joining points were identified using pairwise geometric overlap criteria, which we implemented using an efficient hashing algorithm. With this protocol, we generated peptide backbones that we then ranked by multiple criteria, filtered, and used as structural templates for sequence design. A detailed description of each step in the computational design protocol is provided in the methods.

### 2.2 Peptides from solved complexes are covered by seeds

To explore the feasibility of designing new binders from seeds, we first asked whether seeds generated from single‐chain structures could be used to cover known peptide binding structures. Specifically, we tested whether native peptide binding modes from 486 structures of peptide‐protein complexes (PixelDB‐486) could be covered by seeds generated from a database of single‐chain structures (see methods). For each peptide‐protein complex, we removed the peptide atoms and generated seeds around each residue in the binding site and then examined whether the seeds overlapped the native peptide binders. This test provided a means to explore how the ability to generate target‐complementing seeds depends on the protein binding‐site structure and the details of the search procedure.

The number of seeds that can be generated depends on the binding‐site fragment that is used for the structural search. Large, complex binding‐site fragments capture more of the structural context of the binding site, but can be less common in other protein structures. To balance this tradeoff, we used a greedy algorithm to find, for each binding‐site residue, the most complex binding‐site fragment that returned at least *N* structural matches (Figure S1). We generated fragments for a range of *N* (500–100,000) matches. When *N* was 5,000 or larger, the median fragment size was just 3 residues. Only when relatively few matches were required did we observe larger fragments: when *N* = 2,500, the median size was 5 residues, and when *N* = 500, the median size was 9 residues (Figure S2). In general, large fragments consisted primarily of α‐helix/β‐strand secondary structure. With the goal of generating many seeds with diverse structures, we set *N* = 100,000 for most of the tests described here (unless otherwise indicated) and used a simplified approach to define *single‐segment* target‐protein fragments ranging in size from 3 to 7 residues (see Supplementary Methods).

Binding‐site fragments differed considerably in the number of seed residues that could be generated from their structural matches. The average number of seed residues generated per match in the single‐chain database was 5.2 ± 4.0 *SD*. As expected, the structural context around a binding‐site residue was an important factor in the number of seed residues that could be placed into the context of the target structure. Binding‐site residues that were more buried by other protein residues generated fewer seeds (Figure S3). We tested whether a database of multi‐chain structures would provide more seeds than the single‐chain database, but this only modestly increased the average number of seed residues per match to 5.8 ± 3.7 *SD* (Figure S4).

**FIGURE 2:**
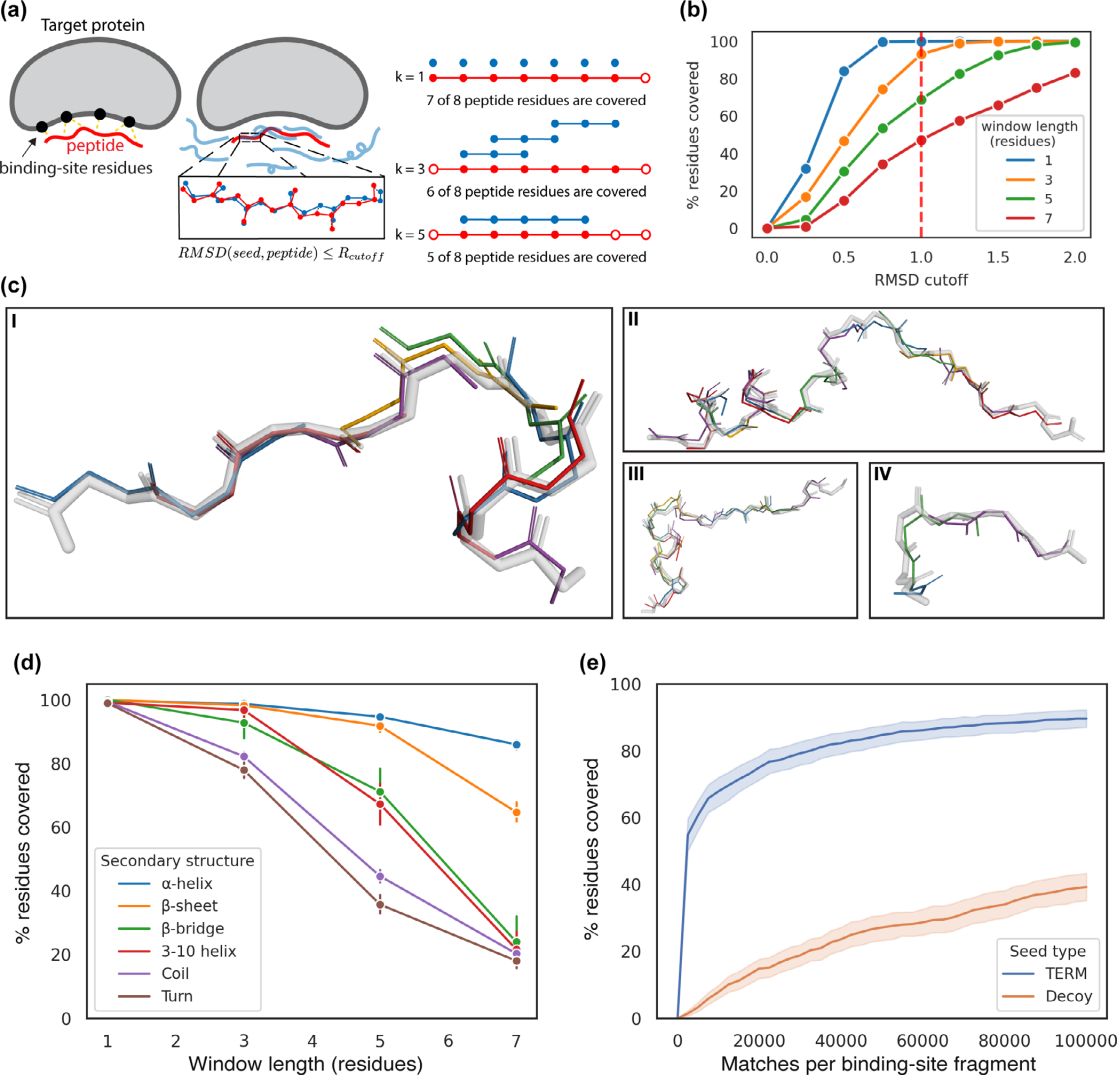
Peptides from known structures are covered by seeds (a) Schematic illustrating the coverage benchmark. The peptide and seeds are shown in red and blue, respectively. Variable‐length alignments are identified between seeds and the peptide and used to determine which peptide residues are covered. (b) The percentage of peptide interface residues covered over a range of RMSD cutoffs is shown for different window lengths. The dashed red line indicates an RMSD cutoff of 1.0 Å. (c) Examples of peptides and the seed windows covering them. For four peptides from experimental structures, the lowest RMSD seed window covering each three‐residue window of the peptide is shown, unless no seed was found to cover that window. The native peptide is rendered in transparent grey and the seeds are colored. The protein binding partner is omitted for clarity. Coverage was defined with an RMSD cutoff of 1.0 Å. (d) The percentage of peptide interface residues covered by seeds over a range of window lengths. The peptide interface residues are grouped and colored by their secondary structure. Coverage was defined with an RMSD cutoff of 1.0 Å. The bars indicate 95% confidence intervals computed by bootstrapping. (e) The percentage of peptide interface residues that are covered based on the number of matches per binding site fragment, for TERM seeds (blue) and decoy seeds (orange). Coverage was defined over 3‐residue windows with an RMSD cutoff of 1.0 Å. The shaded regions indicate 95% confidence intervals computed by bootstrapping

Most native peptide structures were recovered by seeds. For each peptide, we searched for all *k*‐residue alignments between the peptide and seeds generated around the binding site. If a seed was found to align to a *k*‐residue window of a peptide, then those residues were considered covered (Figure 2a). Of the 7,480 peptide residues in the PixelDB‐486 test set, we defined 4,998 with the potential to contact the target protein as *peptide interface residues* (see methods). We focused our analysis on coverage of peptide interface residues, calculating coverage over a range of alignment lengths and backbone atom RMSD cutoffs to understand broader trends (Figure 2b). When considering single residues of the peptides (*k* = 1), all peptide interface residues were covered at an RMSD cutoff over all four backbone atoms of 1.0 Å, and even at the more stringent cutoff of 0.75 Å. With window size *k* = 3, seeds still covered nearly all residues with an RMSD cutoff of 1.0 Å. At longer alignment lengths, (*k* = 5 or 7) we found that coverage at an RMSD cutoff of 1.0 Å dropped to 63% or 42%, respectively. Visual inspection confirmed that the seeds recapitulated the structural features of the peptide regions they overlapped. For window length 3 (RMSD cutoff of 1.0 Å), we compared the structure of each peptide to the best overlapping 3‐residue seed window and confirmed that the alignment reflected high structural similarity, as shown in Figure 2c.

The degree to which peptides were covered, particularly at longer window lengths, depended on the secondary structure of the peptide (Figure 2d). While residues of all secondary structures were at least 80% covered at a window length of 3 residues, at longer window lengths, coverage dropped substantially for all structure types except α‐helix and β‐strand. For example, at *k* = 7, α‐helix and β‐strand residues were still ~90% and ~70% covered, respectively, whereas other secondary structures were close to 20% covered. We assigned secondary structure to the seed residues and found that the composition varied among binding sites (Figure S5). When considering seeds generated around all binding sites, we found that seed residues had a higher proportion of α‐helix and β‐strand structures and less coil relative to the peptides in the dataset. The overall distribution of seed secondary structures was closer to the distribution of the structural database from which they were generated (Figure S6).

Our initial evaluation of coverage used 100,000 matches per binding‐site fragment, but further analysis showed that a much smaller number of matches could generate seeds that cover the majority of peptide interface residues (Figure 2e). We ranked binding‐site fragment matches by RMSD and sequentially added the seeds that they generated to the set used for coverage. We found that the top 2,500 matches were sufficient to generate seeds that cover 58% of the residues with 3‐residue windows and an RMSD cutoff of 1.0 Å. As we considered more matches, the degree of coverage increased, but at a diminishing rate. The residue types that were best covered with relatively few matches were β‐strand, β‐bridge, and α‐helix residues (Figure S7), consistent with our finding that these secondary structure types are easier to cover with seeds. We tested whether seeds generated from complex, multi‐segment query fragments could cover more native peptide residues, however, when considering equivalent numbers of matches, greater fragment complexity did not provide any advantage (Figure S8).

As a control for covering peptides trivially by extensive seed sampling, we examined coverage using decoy seeds generated by randomly placing segments of protein backbone around the target protein binding site. The decoy seeds matched the TERM‐generated seeds in number of residues and distance‐to‐protein distribution, but were not generated from database matches (see Supplementary Methods). The decoy seeds covered peptide structures much less efficiently than the TERM‐generated seeds (*k* = 3 residues and RMSD = 1.0 Å, Figure 2e). This effect was particularly pronounced when considering fewer matches per binding‐site fragment. As the number of matches per binding fragment was increased, decoy seeds did not exhibit the initial jump in coverage observed when using TERM‐generated seeds, and instead, the degree of coverage rose steadily with the number of seeds.

### 2.3 Seeds can be fused to reconstruct peptides from solved complexes

**FIGURE 3:**
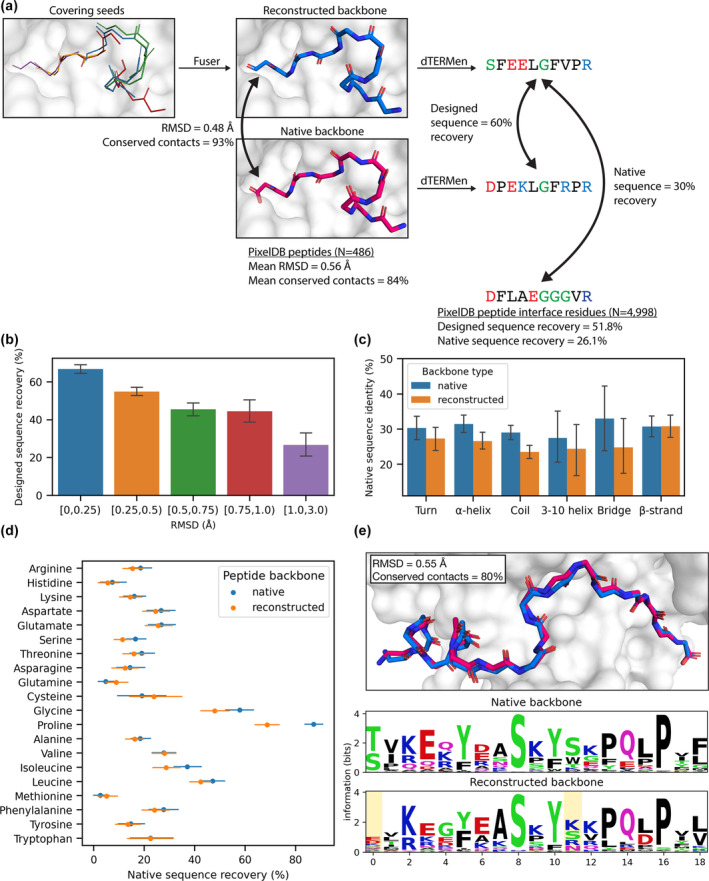
Native peptide structures can be reconstructed by fusing seeds (a) Overview of the native peptide reconstruction process (example native PDB: 1BBR). The RMSD and percentage of conserved potential contacts between the native peptide backbone and the reconstruction are reported. Results are reported for the selected example, as well as the average over all 486 native peptides and 4,998 peptide interface residues. (b) Reconstructed peptide interface residues are binned by RMSD to the native and the average designed sequence recovery is reported per bin. (c) Peptide interface residues are grouped by STRIDE secondary structure classification of the native residue and the average native sequence identity is reported for both the reconstructed and native residues. (d) The percent native sequence recovery on the native and reconstructed backbones is reported for each amino acid. (e) Top: The selected native peptide and the corresponding reconstruction are shown in red and blue, respectively (native PDB: 3QAM). The RMSD and conserved potential contacts between the native and reconstructed backbone are reported. Bottom: sequence logos were constructed from sequences sampled from the native and reconstructed backbone dTERMen energy tables (see methods). Highlighted positions indicate residues in the reconstructed backbone with an RMSD to the native of 0.75 Å or greater. (b, c, d) Error bars indicate the 95% confidence interval estimated by bootstrapping

When applying this approach to protein design, seeds need to be combined to form longer chains. To test whether the relatively short seed windows that cover a native peptide can be joined to accurately recapitulate the structure of known peptide binders, we developed a reconstruction test (Figure 3a). We combined seeds using Fuser, a protocol that takes a set of aligned fragments as input and joins them to generate a new backbone (see Supplementary Methods). We used seeds generated from single‐segment fragments with *N* = 100,000 matches per binding site fragment. For each 3‐residue window of every peptide, we found the corresponding aligned seed window with the lowest RMSD, and provided these segments to Fuser along with the structure of the target‐protein binding site. The vast majority of the peptide interface residues were covered by some seed: only 9 peptide residues out of 4,998 peptide interface residues were not covered and therefore could not be reconstructed.

Peptides reconstructed by fusing seeds had high structural similarity to the corresponding native peptide. Visual inspection of the reconstructed backbones showed that they overlapped the native structure very closely, with small differences in the precise orientation of the residues (Figure 3a,e). The average RMSD difference between the backbone atoms of native peptides and their corresponding reconstructions was 0.56 Å (95% CI [0.55,0.57]), indicating that the peptides were reconstructed with sub‐atomic accuracy. Outlier positions with RMSD >1.0 Å mainly corresponded to residues with coil or turn secondary structures (Figure S9). We also used native contact recovery as a measure of binding mode conservation. The reconstructed peptides had no sequence, so we used a definition of potential contacts that only requires backbone coordinates.^24^ We defined potential contacts between the residues of each native peptide and the interacting protein and then counted how many of these potential contacts were conserved in the corresponding reconstruction. We found that on average 83.7% (95% CI [82.9,84.4]) of native potential contacts were conserved by the reconstructed backbones, supporting that the binding poses of the reconstructed backbones were very similar to their native counterparts.

To complement our structural analysis, we used dTERMen to design a sequence for each reconstructed backbone^25^ and compared this to 1) the dTERMen‐designed sequence for the corresponding native backbone (the “designed‐sequence recovery”) and 2) the native sequence (the “native‐sequence recovery”) (Figure 3a, see methods).^35^ The designed‐sequence recovery was 51.8% (95% CI [50.7,53.0]). This is slightly higher than the value obtained when dTERMen was used to design sequences on distinct models from high‐quality NMR ensembles (47%),^25^ indicating that the reconstructions are within the range of native protein structural fluctuation. As expected, reconstructed residues with very low RMSD to their corresponding native residue had an even higher recovery, while residues with RMSD of 1.0 Å or greater had lower sequence recovery (Figure 3b).

The native‐sequence recovery, computed only over peptide interface residues, was 26.1% (95% CI [25.0,27.1]). This was slightly lower than the same value when designing on the native backbone, which was 30.1% (95% CI [29.1,31.3]). The native‐sequence recovery varied by the secondary structure of the native residue. Residues of all secondary structures, except β‐strand, showed a small drop between the native and reconstructed backbones (Figure 3c). The native‐sequence recovery of reconstructed β‐strand residues was almost the same as the native backbone, consistent with those reconstructed residues having low RMSD to the native residues (Figure S9). We grouped the native peptide positions by their residue identity and found that the rate of recovery did not show large biases (Figure 3d). The two exceptions were glycine and proline, which showed 18% and 10% lower sequence recovery on the reconstructed backbones vs. native backbones. At positions where glycine and proline were recovered on the native but not the reconstructed backbone, we observed a higher RMSD difference between reconstructed and native backbones (Figure S10), consistent with differences in sequence recovery tracking with structural deviations, as expected.

To look at broader trends, we used Markov Chain Monte Carlo sampling to generate ensembles of sequences predicted by dTERMen to be compatible with each reconstructed backbone and represented them as sequence logos (methods, Figure 3e). The sequence logos reflected the high degree of sequence conservation between the native and reconstructed backbones; the consensus sequence of native and reconstructed interface residues had a 55.7% (95% CI [54.7,57.0]) identity. Native peptide positions varied in their information content, with some positions favoring a single amino acid and others allowing a variety of amino acids. The per‐position sequence conservation was correlated between the native and reconstructed backbones (Pearson *R* = 0.66), indicating that many positions with strong amino‐acid preferences in the native backbone context also had strong preferences in the reconstructed backbone (Figure S11). Of the 560 positions in the native peptides where a single amino acid was highly favored (sequence conservation ≥4 bits), 497 or 88.7% of the corresponding positions in the reconstructed backbones had the same amino acid.

### 2.4 Designing peptide binders of TRAF6

To explore how this approach could be applied to design protein‐binding peptides de novo, we conducted a case study that involved generating seeds around an entire protein domain surface and using our method to sample de novo backbones on which we designed sequences. Our target was tumor necrosis factor (TNF) receptor‐associated factor 6 (TRAF6), a human scaffolding protein with important roles in signal transduction.^36^ We focused on the TRAF domain, which binds to the cytoplasmic tails of cell‐surface receptors. Inhibiting the protein interactions of this domain has been associated with decreased osteoclastogenesis,^37^ increased insulin sensitivity,^38^ and reduced breast cancer metastasis.^39^ Structures of TRAF6 in complex with natural peptide ligands have revealed a conserved binding mode.^40^

**FIGURE 4:**
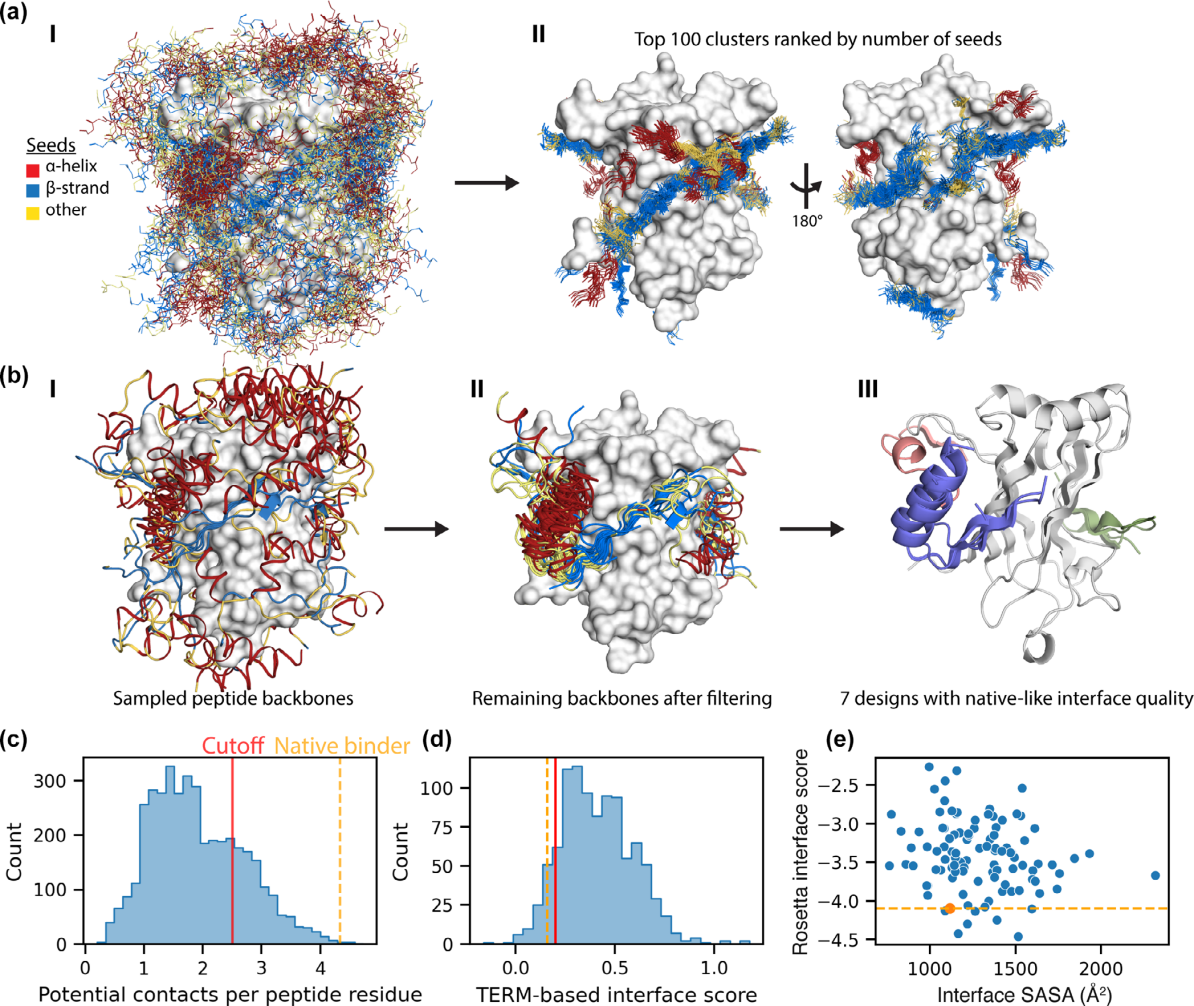
Designing peptide binders of TRAF6 (a) Seeds generated around the surface of TRAF. (I) 1,000 seeds randomly sampled for visualization. (II) The 100 most common seed binding modes. Seeds are colored according to their secondary structure. (b) Sampling and filtering peptide backbones to obtain a final set of designs. (I) Representative sampled backbones from 70 peptide backbone clusters. (II) The remaining peptide backbones after filtering by the number of potential contacts per residue and TERM interface score. (III) Designs with better normalized Rosetta energy of binding than the native binder CD40. (c) The distribution of potential contacts per peptide residue for 4,000 sampled peptide backbones. (d) The distribution of TERM interface scores for 986 backbone designs, after filtering based on potential contacts. (e) The Rosetta energy of binding, normalized by the size of the interface, for the final set of 100 sampled backbones after filtering. Designs are shown in blue and CD40 is shown in orange. (c–e) The orange line and dot correspond to the native TRAF6 binder; the red line corresponds to the cutoff value that was used to filter designs at each step

To obtain a structural model of TRAF6 alone, we removed the peptide ligand from the TRAF6‐CD40 structure (PDB ID: 1LB6) and targeted a single, monomeric domain. We generated seeds around the entire surface of the TRAF domain (Figure 4a). We defined single‐segment binding‐site fragments such that each had 5,000 matches in the database; this resulted in a total of 575,000 seeds with a median length of 6 residues. As before, we saw considerable variation in the number of seeds generated by each protein fragment (824–11,464 seeds, Figure S12). Seeds were unevenly distributed around the protein domain (Figure 4a) and we used greedy clustering to identify the most populated binding modes (see methods). The 100 largest clusters included examples of β‐strand seeds pairing with unpaired β‐strands on the protein surface, helices packing against the β‐sheets, and a few scattered clusters lacking regular secondary structure (Figure 4a).

Many seeds overlapped, suggesting that they could be combined to generate diverse longer peptide structures. We used a stringent overlap definition for fusion, requiring 4‐residue overlaps that satisfied both distance and orientation criteria (see Supplementary Methods). In total, we found 19 million overlaps, which were distributed very unevenly over seeds (Figure S13). 60% of seeds overlapped no other seed, while some seeds overlapped as many as 10,000 other seeds. Seeds with many overlaps were overwhelmingly β‐strand‐pairing seeds. We constructed a seed graph and sampled random paths until we obtained 4,000 paths that satisfied our criteria (see methods). Of 66,671 paths sampled, the vast majority (~93%) were rejected because they were shorter than the minimum length requirement of 15 residues (Table S1). Only 0.3% of all sampled paths were identical to a previously accepted path, indicating that further sampling could yield additional unique paths.

We fused the seeds in each of the 4,000 sampled paths that passed our filters to generate peptide backbones and observed many distinct binding modes. Using a permissive criterion for similarity (20 Å RMSD), we defined 70 clusters, which we visualized by selecting cluster representatives (Figure 4b). The representative peptide backbones spanned the surface of TRAF6, demonstrating that designs are generated at many distinct sites. Representatives had predominantly helical or strand secondary structure composition, but also included loops connecting these elements. We also clustered with a tighter threshold of 5 Å and observed 1,373 clusters, indicating that many of the designed peptide backbones represented variations on a similar binding mode.

We compared our designed peptide backbones to native peptide backbones using contact‐based criteria and used these to identify the most promising design candidates. Examining the potential for residues in the peptide to contact the protein, we found that native peptides (PixelDB‐30, see methods) had a median of 4.2 potential contacts per residue, although this value was lower for peptides with 15 or more residues, for which the median was 3.4 potential contacts per residue with a range of 2.4–5.5 (Figure S14). The designed backbones had a much lower median: 1.8 potential contacts per residue. Nonetheless, the designs included many examples with as many potential contacts per residue as native peptides, and we filtered designs using a permissive cutoff of 2.5 potential contacts per residue, which yielded 986 backbones (Figures 4c and S15). Next, we applied a TERM interface score that quantified the compatibility between the designed backbone and the protein surface (see methods). This score ranged from −0.29 to 0.92 for native peptides, with negative scores being more favorable, with a median of 0.08 (Figure S16). The native TRAF binder, CD40, had a score of 0.16, which we used to define a slightly more permissive cutoff of 0.2, resulting in 108 backbone designs (Figure 4d). To further reduce this number prior to the next stage, we looked at the clusters defined at an RMSD of 5.0 Å and selected up to the top three best‐scoring backbone designs from each cluster, providing 100 backbone designs from the 70 clusters (Figure 4b).

We used dTERMen to design sequences on the final set of selected backbones and used Rosetta Relax to construct structural models (see methods). We scored the models of the designs, along with a set of native peptide‐protein complexes subjected to Rosetta Relax (PixelDB‐30), with the Rosetta Interface analyzer.^41^ This approximates the binding energy by computing the change in Rosetta energy when separated binding partners are brought together to form a complex, with no change between the bound and unbound monomer structures. We used the surface area‐normalized Rosetta interface score to rank the quality of designed interfaces and to compare them to the native CD40 binder interface (Figure 4e). The mean Rosetta interface score for the 100 designs was −3.4 ± 0.45 SD, which was slightly less favorable than native peptides (−3.8 ± 0.64 SD, Figure S17). We also computed the packstat score, a measure of interface packing quality,^42^ and found the average values were identical for the designs and native peptides (0.62, PixelDB‐30, Figure S18).

**FIGURE 5:**
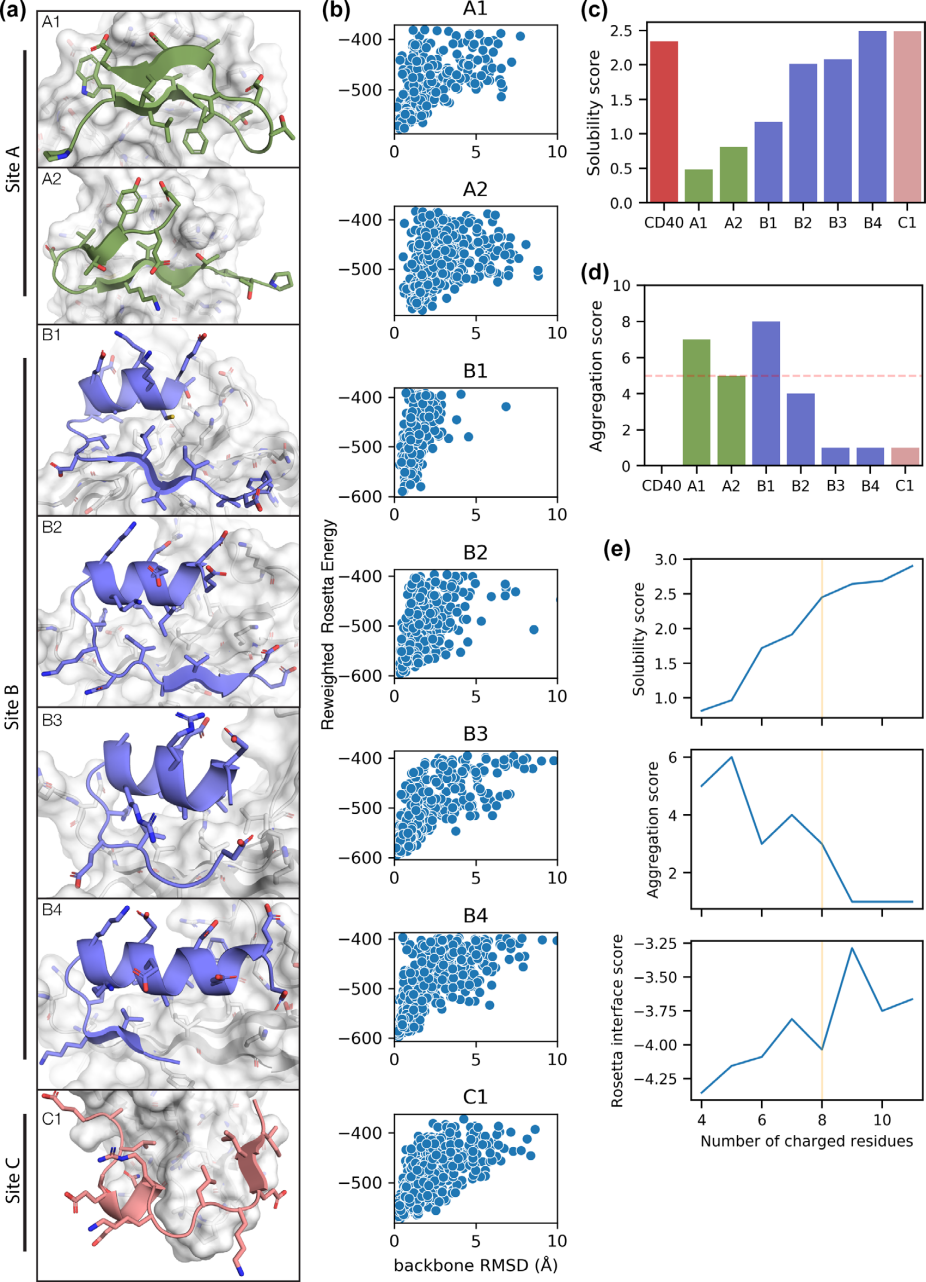
Characteristics of top‐ranked peptide designs (a) Selected designs interact with three distinct sites on TRAF. The designs are colored based on which TRAF site they engage. (b) The reweighted Rosetta Energy vs. backbone RMSD of peptide structural models sampled with FlexPepDock Refine. (c) The CamSol solubility score calculated for CD40 and the designs. (d) The AMYL2PRED aggregation score calculated for CD40 and the designs. The threshold used to classify peptides as aggregation‐prone is shown by the dashed red line. (e) The CamSol solubility score, AMYL2PRED aggregation score, and Rosetta interface energy are shown for A2 sequences designed with varying numbers of charged residues. The yellow line highlights the number of charged residues at which aggregation potential is decreased and the Rosetta interface score is close to the original A2 design

We focused on seven designs that gave a more favorable Rosetta interface score for binding to TRAF6 than a native peptide binder from CD40 (see Supplementary Table S2). These designs interacted with three sites: A) the native binding site (Figure 5a, green), B) an exposed β‐strand and sheet on the other side of the protein (Figure 5a, purple), and C) a loop (Figure 5a, pink). Two of the high‐scoring designs engaged the native binding site (site A). Both paired with the exposed β‐strand and included a second anti‐parallel β‐strand, but with different turns connecting the second β‐strand to the first. Four designs interacted with site B; each design had the same α‐β topology, with the β‐strand pairing with an exposed TRAF6 β‐strand. The designs differed in the length of the β‐strand pairing interaction, the packing of the helix, and the turn connecting the two structural elements. One design interacted with site C, a TRAF6 loop. The design consisted of a short helix and a β‐strand connected by a loop.

The best scoring designed peptides had properties similar to those of native peptide binders. The top seven designs formed between 1.2 and 1.6 hydrogen bonds per 100 Å^2^ of buried interface surface area. This was slightly less than CD40, which forms 1.7 per 100 Å^2^, but is in line with the average value observed for native peptides (1.1 ± 0.6 *SD*, PixelDB‐30). The TERM interface scores ranged from 0.16 to 0.19, with the exception of design C1, which interacted with a considerably more favorable score of 0.03. These scores were close to those for native peptides (0.11 ± 0.23, PixelDB‐30). Finally, the packstat of the designs ranged from 0.62–0.68, which was similar to the native peptides (0.63 ± 0.09, PixelDB‐30). All of the statistics computed for the top seven designs and CD40 are provided in Table 1.

**TABLE 1:** Properties of designed peptide binders of TRAF6

Design	Length (residues)	Potential contacts per residue	Hydrogen bonds per 100 Å^2^	TERM interface score	Rosetta interface score	Packstat
A1	15	3.0	1.3	0.16	−4.1	0.64
A2	15	3.3	1.2	0.19	−4.3	0.68
B1	20	3.6	1.5	0.18	−4.1	0.68
B2	20	3.0	1.6	0.16	−4.6	0.62
B3	17	2.6	1.5	0.16	−4.1	0.62
B4	20	2.5	1.4	0.17	−4.2	0.62
C1	16	2.8	1.5	0.03	−4.4	0.64
CD40 (native)	9	4.3	1.7	0.16	−4.1	0.71

To assess whether the top‐ranked designed TRAF6‐peptide complexes are predicted to lie in energy minima, we used the FlexPepDock Refinement (FPD Refine) protocol to explore the local energy landscape for each structure.^43^ Six of the top designs had a single local energy minimum within a backbone RMSD of ~0.5 Å with respect to the relaxed structure (Figure 5b). Design A2 also showed a single energy minimum, albeit at a backbone RMSD of 1.7 Å from the relaxed structure. The differences between the relaxed and FPD Refine minimum‐energy structural models of A2 were primarily at the termini (Figure S19). Altogether, computational analysis supports that the binding interfaces formed by seven top‐scoring designs are enthalpically favorable and that the designed binding mode is predicted to be stable according to the Rosetta energy function.

We also considered whether the peptide designs are predicted to form off‐target structures or aggregates when unbound. Deep learning methods such as AlphaFold and RoseTTAfold provide highly accurate structure predictions for many native proteins, and although the methods make use of evolutionary information, recent work has demonstrated that they can accurately predict the structures of *de‐novo* designed proteins that adopt stable folds. Such methods also assign low confidence to residues that correspond to intrinsically disordered regions.^44^, ^45^ We used ColabFold to predict the structure of each of the seven designed peptides in the absence of the target protein.^46^ Interestingly, all of the peptides were predicted to form structures with at least moderate confidence (77–96 pLDDT, Figure S20), and AlphaFold models for four of the designs (A2, B1, B2, and B4) were structurally similar to the designed conformation, with full atom RMSDs ranging from 2.0–3.2 Å (Figure S20). Peptide A1 was predicted to consist primarily of β‐strand secondary structure, as designed. Although the top‐ranked model for peptide B3 lacked the β‐strand, the third‐ranked model, which had a similar mean pLDDT (84.6 vs. 85.3), displayed the same α‐β topology as the designed structure (Figure S21). The structure of design C1 was predicted to be mostly α‐helical, with high confidence (pLDDT 96%). While the models had moderately high mean pLDDT values, the pTM was consistently low (0.03–0.08), indicating that AF2 was more confident in predicting local features of the peptides, such as the secondary structure, rather than the overall structure. This is unsurprising, given that it is unlikely that any of these short peptides form stable structures when unbound. Nevertheless, the AlphaFold predictions indicate that sequences designed to interact with sites A and B are well suited for their intended binding conformations.

To evaluate whether the high‐scoring designed peptides have a propensity to form non‐specific aggregates, we used CamSol, a sequence‐based statistical method for predicting solubility.^47^ The CamSol solubility score is scaled so that random peptides from the human proteome have a mean score of 0 and a standard deviation of ±1. Two of the designed peptides, B4 and C1, were predicted to have higher solubilities than CD40 (Figure 5c). B2, and B3, were also predicted to be soluble, but less so than CD40. Designs A1, A2, and B1 were predicted to have the lowest solubility scores, but they were still positive . We also considered the possibility that the designed peptides could aggregate by forming amyloid‐fibril structures. We assessed the potential for each of the seven designs to form amyloid aggregates using AMYL2PRED, a consensus prediction method that scores a sequence as aggregation‐prone if half or more of the underlying algorithms (five, in this case) consider the sequence to be aggregation‐prone.^48^ Three of the peptides, B3, B4, and C1, were only predicted by one of 10 algorithms to be prone to amyloid aggregation (Figure 5d). Peptide B4 was predicted by four of 10 algorithms to be prone to forming aggregates, making it an intermediate case. Three of the peptides, A1, A2, and B1, were predicted by more than half of the algorithms to aggregate. We considered the per‐residue solubility/aggregation scores for designs A1, A2, and B1, and found that, as expected, hydrophobic residues within β‐strands were predicted to contribute to low predicted solubility and higher potential for aggregation (Figure S22).

We explored whether peptides interacting with site A could be designed to be less aggregation‐prone. We reasoned that increasing the number of charged residues would make the peptides more soluble and protect them from forming off‐target β‐strand pairing interactions in amyloid fibrils. Taking advantage of dTERMen, which defines the energy landscape as a linear function of sequence and pre‐computed energy parameters, we used integer linear programming to optimize the energy of the A2 peptide subject to a constraint on the number of charged residues (see methods). We repeated the energy optimization multiple times, incrementing the number of charged residues, and scored the designed sequences for amyloid‐like aggregation and interface quality (Figure 5e). As expected, the solubility score increased with the number of charged residues. Likewise, the aggregation score dropped as the number of charged residues increased, but the trend was not strictly monotonic. The Rosetta interface energy became less negative as the number of charged residues in the peptide increased, reflecting a trade‐off between predicted binding affinity and aggregation propensity. Notably, the sequence obtained when requiring 8 charged residues (four more than the originally designed sequence) had a significantly higher solubility score and lower aggregation score, with only a relatively small drop in Rosetta interface score (Figure 5e).

## 3 DISCUSSION

Despite numerous advances in computational methodology, it remains difficult to design binders of a protein target de novo. The majority of successful studies have approached this task using repurposed protein scaffolds,^5^, ^14^, ^16^ effectively prioritizing the stability of the binding protein over the formation of an optimal interface with the target. An alternative function‐guided approach is to design the binder to be complementary to the desired protein target binding site. Our work shows that TERMs from single‐chain structures in the PDB can be used as modular building blocks to construct target‐complementing interface structures. In a test of the feasibility of this approach, we found that TERMs can be applied to reconstruct the structures of known peptide ligands that exhibit broad structural diversity. As a proof‐of‐concept for application in design, we demonstrated a computational method that uses TERMs to generate new peptide backbones that complement both known and new binding sites on a target protein with interface quality equivalent to a native peptide binder. Coupled with sequence‐design algorithms, this approach has potential for de novo peptide binder design.

Other work has explored the question of whether peptide‐protein and protein–protein interfaces exhibit structural similarities to protein cores. Vanhee et al. defined pairwise interacting fragments at known peptide‐protein interfaces, similar to TERMs, and found that matching fragments could be found in monomeric proteins.^49^ We demonstrated that we can start with the target protein structure alone, rather than a pre‐defined interface, and identify TERMs that can be aligned to the target to define seeds that cover native peptide binders. Hadarovich et al. recently compared fragments from protein–protein interfaces and protein cores and found that they are largely different.^50^ The discrepancy between this result and our finding, as well as that of Vanhee et al., can be explained by how structural fragments were defined in each case. The fragments used by Hadarovich et al. encompass large portions of the interface, with each fragment consisting of hundreds of residues. We use significantly smaller structural motifs, which recur at high frequency in the PDB, but are still large enough to capture important structural features of interfaces. Interestingly, even three‐residue binding‐site fragments can generate seeds that cover significantly more peptide residues than decoy seeds with randomly determined poses (Figure 2e).

Other groups have applied TERMs to the structural search problem in peptide‐protein docking^28^, ^29^ and design.^30^ For example, the PatchMAN docking method threads a known peptide sequence onto multiple TERM‐derived backbone fragments and then uses FlexPepDock Refine to fine‐tune the structures and evaluate their energies. The design method PepComposer samples short TERM‐like fragments that complement a protein surface and performs alternating cycles of sequence design and relaxation to design the peptide binder. The fragments that are used are short and have only local complementarity to part of the target, limiting their applicability to designing peptides ~7 residues or shorter. In this work, we showed that a great diversity of longer backbones can be generated by combinatorial assembly, facilitating the discovery of diverse structures that complement the target over a greater area. This is analogous to combining structural elements to build complete protein folds using SEWING.^34^

Building larger interfaces from small, TERM‐derived seeds requires a protocol for assembling seeds into longer chains with realistic stereochemistry. We showed that seeds with geometric overlap can be combined by Fuser to reconstruct native peptide backbones and to build novel candidate peptide backbones. Importantly, we found that the designed‐sequence recovery of reconstructed peptide backbones was high, indicating that the reconstructions accurately recapitulated structural features that are relevant to the sequence design algorithm dTERMen. Peptide residues with β‐strand or α‐helix secondary structure were reconstructed with the highest accuracy, but we were also able to reconstruct other secondary structure types such as turn and coil (Figure S9). Our seven highest‐ranking de novo peptide designs for binding to TRAF6 included turns or coil segments connecting β‐strand and α‐helix elements (Figure 5a). Thus, although TERMs may be most readily applied to designing interfaces featuring β‐strand or α‐helix secondary structures, they are not limited to these structures.

We explored the ability of our method to generate novel, physically realistic peptide binders by designing peptides to interact with the TRAF domain of the protein TRAF6. The Rosetta interface score and packing statistics of the final set of 100 designs were very similar to those for known peptide‐protein complexes, suggesting that in these respects the designed interfaces are of similar quality to naturally evolved ones. Further in silico characterization by FPD Refine of the seven top‐ranked designs indicated their binding modes are stable. While it is difficult to rule out undesired off‐target conformations, AlphaFold‐predicted structures for the unbound peptide monomers did not reveal other highly favored states and were remarkably similar to the intended conformation for more than half of the designs, indicating good sequence‐structure compatibility. While some of the designed peptides were predicted to be prone to aggregation, requiring more charged residues during sequence design reduced this liability. We observed considerable structural diversity among the designs, with many targeting new sites on TRAF6. We sampled a small fraction of the total number of paths in the graph, and more extensive sampling would likely provide new binding modes or variations on those already observed. Many of the designs exhibit N and C termini in close proximity, making them candidates for cyclization. Another promising direction is to expand the peptides into mini‐proteins by designing a scaffold to present the designed interface.^51^, ^52^ This approach would represent a marriage of existing methods, where the interface is designed to prioritize target complementarity and then subsequently supported by structural elements that promote folding and stability.

In this work, we have demonstrated the utility of TERMs for the design of novel binders. The evidence presented here supports that TERMs have broad applicability across all known peptide types, but are most immediately applied to designing binders with α‐helix and/or β‐strand structures. Our computational design method can be used to generate novel putative peptide binding structures with high complementarity to an intended binding site, a task which is not easily achieved by existing methods.

## 4 MATERIALS AND METHODS

A detailed description of the peptide design method is provided in the Supporting Information.

### 4.1 Large set of non‐redundant peptide‐protein complex structures (PixelDB‐486)

The dataset used for peptide coverage and reconstruction was created from PixelDB, a database of 1,966 high‐quality peptide‐protein structures.^31^ The complexes in PixelDB are grouped by the structure of the peptide‐bound protein, resulting in 486 clusters. From each cluster, we selected the complex with the peptide with the lowest average B‐factor (see Table S4). For each complex, we defined the binding site as the set of protein residues that make at least one potential contact of any type with the peptide. Since the sequence of the protein is fixed, we modified the contact definition protocol slightly, such that only rotamers of the native amino acid were considered at positions within the protein chain. We defined peptide interface residues as those residues that (1) make at least one of the previously defined potential contacts that define the target protein binding site and (2) have a B‐factor within three standard deviations of the mean B‐factor over all atoms in that complex. There were 7,480 residues total in the peptides, of which 4,998 were defined as interface residues. We refer to this set as PixelDB‐486.

### 4.2 Native peptide coverage benchmark

We defined a native peptide as *covered* if all peptide residues are well‐aligned with at least one seed. Consider native peptide p and seed s. We define a *k*‐residue alignment starting at residue i in p and j in s if the backbone‐atom RMSD between segment $\left(p_{i},p_{i+1},\ldots ,p_{i+k-1}\right)$ and segment $\left(s,s_{i+1},\ldots ,s_{i+k-1}\right)$ is $\leq R_{c}$. If this condition is satisfied, all residues in $\left(p_{i},p_{i+1},\ldots ,p_{i+k-1}\right)$ are considered covered. No superposition is applied in this process; seed atoms are fixed in space. For each value of k, we compare all *k*‐residue windows of the peptide with all *k*‐residue windows of all seeds. In this work, we used *k* = 1, 3, 4, 5 or 7 residues and tested different values of *R*_*c*_ in the range *R_c_* = [0.0, 3.0] Å.

### 4.3 Identifying contacting residues

Throughout this work, we used potential contacts, which capture the potential for two residues to participate in an interaction given knowledge of just the backbone atoms of the protein (i.e., without considering the sequence). The types of contacts we consider include sidechain‐sidechain, sidechain‐backbone, and backbone‐backbone interactions. We used *contact degree* to identify potential interactions mediated by sidechains.^24^ To identify potential sidechain‐backbone interactions, we used *interference* ^25^. Both of these provide a value between [0, 1] for each pair of residues: to obtain a binary criterion for contacts, we applied a threshold of 0.01. Finally, we identified backbone‐backbone interactions by finding all pairs of positions with any inter‐residue backbone atom distance ≤ 3.25 Å.

### 4.4 Assigning secondary structure labels to residues

We used STRIDE to assign secondary structure labels to residues in the structural database.^53^ Residues were labeled in their original structural context and these labels were preserved when residues were copied and placed around the target as seeds. In some cases, STRIDE was unable to classify a residue, for example, if the residue had an uncommon amino‐acid identity. If the flanking residues (+/− 1) had the same STRIDE classification, then this was assumed to be the correct label for the residue in question. In all other cases, the residue was assigned as coil.

### 4.5 Native peptide reconstruction and design benchmark

Each peptide was reconstructed from seeds identified in the coverage benchmark. We used seeds that covered 3‐residue windows with an RMSD cutoff of 2.0 Å. This provided a set of seed windows covering most peptide windows. For each window, if there were multiple seeds aligned, we selected the seed with the lowest RMSD to the peptide. We constructed the Fuser topology as described in the Supplementary Methods, with some modifications. If a window of the peptide was not covered by a seed, then there was no structural information for that window when fusing, and the position was omitted from the topology. We required that each window of the peptide be covered by a different seed, meaning that all reconstructed backbones were chimeric structures. After selecting the set of seeds covering each three‐residue window of the peptide, we added the structure of the target protein and each seed window with its corresponding anchor to the topology and performed fusion to generate the reconstructed peptide backbone.

To generate an ensemble of low‐energy sequences for the native and reconstructed peptide backbones, we used a modified version of the Markov chain Monte Carlo simulated annealing (MCMC SA) protocol used in de novo design (see Supplementary Methods) for sampling sequences given a dTERMen energy table. To account for the fact that the dTERMen energy scale varies between structures, we adjusted the final temperature used when sampling sequences by MCMC SA. To find the final temperature, we first estimated the standard deviation of each energy table, s, and then computed $E=E_{\min}+\frac{1}{2}s$. We repeated the sampling process, scanning over a range of final temperature values, and selected sequences sampled at a final temperature with an average energy closest to E. The sequence conservation at each position was calculated by subtracting the entropy of residues observed at that position from the maximum possible entropy. The sequence logos resulting from this procedure displayed a range of amino‐acid conservation: some positions were highly variable, while others still displayed a strong preference for certain amino acids. We defined a consensus sequence for a given sequence logo by selecting the highest probability amino‐acid residue at each position in the peptide.

### 4.6 Designing peptide binders of TRAF6

We used an experimentally determined structure of TRAF6‐CD40 complex (PDB ID: 1LB6,^40^ CD40 peptide removed) to model the TRAF domain. We attempted to generate seeds from single‐segment fragments (*m* = 3) around every residue in the protein. The number of seeds generated per TRAF6 residue is reported in Figure S12 for surface residues (those with relative solvent accessible surface area > 0.05), as calculated with FreeSASA.^54^ We searched for pairwise overlaps between seeds, constructed a graph, and sampled 4,000 paths as described in the Supplementary Methods. We rejected paths sampled from the seed graph if they consisted of a seed with no alignment to other seeds, had fewer than 15 residues, exhibited a VDW clash with the protein after fusing (*Equation 1*, Supplementary Methods), or were redundant to a path that had already been accepted. Seeds in paths were fused to yield peptide backbones and, for a subset of the peptide backbones, sequences were designed using dTERMen as described in the Supplementary Methods.

### 4.7 Clustering seed binding modes

We used a simple greedy set cover algorithm to cluster the seed binding modes. We defined U as the set of all *k*‐residue windows of all seeds and found alignment by geometric criteria (*Equations 2*,*3*, Supplementary Methods). We found the *k*‐residue seed window $\left(s_{A},i\right)$ with alignment to the most other seed windows. $\left(s_{A},i\right)$ and the aligned windows were defined as the first cluster (with $\left(s_{A},i\right)$ as the representative), all were removed from U, and process was repeated. We continued until 10% of the seed windows in U were included in some cluster. We used *k* = 4 residues when finding aligned seed windows.

### 4.8 Clustering designed peptide backbones

We performed an all‐to‐all comparison of designed peptide backbones to find their structural similarity by RMSD. If two peptides varied in length, the RMSD was computed between the shorter peptide and all equal length windows of the longer peptide, to find the best alignment. We performed hierarchical clustering with a complete‐linkage criterion to find clusters at varying RMSD thresholds. Cluster representatives were defined as the peptide with the lowest average RMSD to all other peptides in the cluster.

### 4.9 Peptide–protein complex structures selected for Interface analysis (PixelDB‐30)

We manually curated a set of 30 peptide‐protein complexes from PixelDB, which we refer to as PixelDB‐30, to use for comparison of native vs. designed backbones. We started with the largest cluster, selecting the single peptide‐protein complex with the lowest relative B‐factor, and continued to the next largest cluster until we had 30 complexes (see Table S27). We omitted complexes in which the peptide appeared to be an integral part of the protein fold.

### 4.10 Rosetta full‐atom modeling and Interface analyzer

We used Rosetta Relax to obtain structural models of the designed peptides and native peptides in PixelDB‐30. We used default Relax settings, generated 10 structural models of each peptide, and selected the lowest‐energy model.^55^ We then used the Rosetta interface analyzer to compute various statistics for the Rosetta‐relaxed peptide‐protein structural models, including the Rosetta interface score (ΔG_bind_/ΔSASAx100).^41^ Because packstat has an underlying random component, we used the option –packstat_oversample = 100 to increase the accuracy of the statistic. In all applications of Rosetta in this work, we used the standard weights, REF15, and Rosetta 3.10 Linux Release (2018.33.60351).

### 4.11 FlexPepDock refine

We slightly modified the FlexPepDock (FPD) Refine protocol to increase the structural diversity of the peptide models. We found empirically that increasing the number of cycles over which the repulsive/attractive energy terms are ramped (i.e., the outer cycles) also increased the structural space that was explored by FPD Refine. We repeated the protocol three times, generating 200 structural models using either 10, 15, or 20 outer cycles. We combined the 600 total structural models to generate the RMSD versus energy plots. Otherwise, we used default settings, with the exception of the following arguments: –ex1, −ex2aro, −use_input_sc, −lowres_preoptimize. When scoring the peptide models, we used the reweighted Rosetta energy function, which places a stronger emphasis on the interface energy terms.^56^

### 4.12 Predicting the unbound structures of peptide designs with AlphaFold

We used ColabFold to predict the structure of each of the seven designed peptides in the absence of the target protein.^46^ As ColabFold generates five models for a given sequence by varying the random seed, we selected the model with the highest mean pLDDT, a metric of structural confidence, for further analysis.

### 4.13 Solubility and aggregation predictions

We used the CamSol webserver to calculate the intrinsic solubility of each peptide sequence at pH 7 (https://www-cohsoftware.ch.cam.ac.uk/index.php/camsolintrinsic). We used the AMYL2PRED webserver to calculate the aggregation potential of each peptide (http://thalis.biol.uoa.gr/AMYLPRED2/). We excluded the “AmyloidMutants” method, as the server hosting it could no longer be reached. The threshold used to classify peptides was taken from the original published work.^48^

### 4.14 Optimizing peptide sequence with charge constraints

We used integer linear programming to optimize the sequence of a peptide, given a constraint on the total number of charged residues. We defined charged residues as arginine, aspartate, glutamate and lysine, excluding histidine as it is uncharged at neutral pH. We used the GLPK LP Solver, v4.55.

## AUTHOR CONTRIBUTIONS

**Sebastian Swanson:** Conceptualization (supporting); data curation (lead); formal analysis (lead); investigation (lead); methodology (lead); project administration (equal); software (equal); validation (lead); visualization (lead); writing – original draft (lead); writing – review and editing (equal). **Venkatesh Sivaraman:** Methodology (supporting); software (equal). **Gevorg Grigoryan:** Conceptualization (equal); funding acquisition (equal); methodology (equal); resources (equal); software (equal); supervision (supporting); writing – review and editing (supporting). **Amy Keating:** Conceptualization (equal); funding acquisition (equal); project administration (equal); resources (equal); supervision (lead); writing – original draft (supporting); writing – review and editing (equal).

## Supporting information


**Appendix S1.** Supporting Information
