# Supplementary material for: Tertiary motifs as building blocks for the design of protein‐binding peptides
Source: Protein Sci. 2022 May 10;31(6):e4322. doi: 10.1002/pro.4322 (PMC9088223; doi:10.1002/pro.4322)
Supplement: Supplementary file 1 — Appendix S1. Supporting Information [file PRO-31-e4322-s001.pdf]

Supplementary Material

Swanson et al., “Tertiary motifs as building blocks for the design of protein-binding peptides”.

Contents

- Supplementary Methods
    - pages 1-9
- Supplementary Figures S1 – S22
    - pages 10-22,
- Supplementary Tables S1 – S3
    - pages 23-24

The following Supplementary tables are provided as separate files:

- Table S4**:** S4_PixelDB486SelectedComplexes.txt
- Table S5: S5_PixelDB30SelectedComplexes.txt
- Table S6: S6_SingleChainDatabase2019-01-22.txt
- Table S7: S7_BiologicalUnitsDatabase2019-01-22.txt

Supplementary Methods

Source code for the pipeline, instructions for installation, and example scripts are provided at https://github.com/swanss/peptide_design.

Defining binding site fragments on the target protein surface

We select a binding site on the target protein by specifying a set of surface residues. In some cases, the set of residues is known (e.g., based on a known binder), whereas in other cases all surface residues are considered potential binding site residues. Each of the selected residues acts as a central residue around which a fragment of the binding site is constructed. We reasoned that each fragment should satisfy two properties: 1) it should be large enough to capture the local structural environment around the central residue and 2) it should be sufficiently common in known protein structures to provide data about potential interactions. We defined multiple protocols to extract binding site fragments from the target protein that satisfy these requirements.

The first protocol extracts fragments that include the local-in-chain residues around the central residue, r_i. The user first selects the required number of structural matches, *N*. A fragment is defined from the central residue and *m* flanking residues on each side (i.e., residues $r_i - m$ through $r_i + m$). The initial fragment is constructed with m = 1, corresponding to a fragment with three residues. We use this fragment as the query in a structural search against a database of known protein structures with some RMSD cutoff, R_{cut} (see below). Searches are conducted using backbone atom coordinates only, in a sequence-independent manner. If we find fewer than *N* matches, we raise R_{cut} until we find sufficient matches, at which point the process completes. If we find more than *N* matches when searching with the initial R_{cut}, we

attempt to generate a longer fragment by incrementing the value of m. If the longer fragment has insufficient matches, we revert to the previous fragment and terminate. If the longer fragment has sufficient matches, we again increment the value of m, until $m > m_{max}$, at which point the process terminates. In this work, we used m_{max} = 3, meaning the maximum number of residues possible in a single segment fragment is $2m_{max} + 1 = 7$. We refer to binding site fragments generated by this procedure as single-segment fragments.

The second protocol extracts fragments that include both local-in-chain and local-in-space residues around the central residue. First, a single-segment fragment is defined around the central residue using the first protocol with m_{max} = 2. Next, the set of residues with the potential to contact r_i, $C(r_i)$, are identified in the protein target structure (defined below). We use a greedy algorithm, similar to the one used by Zhou et.al, to add contacting residues and their flanking residues to the fragment[25]. Briefly, we individually add each residue $r_j \in C(r_i)$ and m residues flanking r_j on each side to yield candidate expanded fragments. We select the expanded fragment with the greatest number of total residues that have at least N matches in the database and then remove r_j from the set $C(r_i)$. In the next round, the remaining residues in $C(r_i)$ are individually added to the current fragment to yield further candidate expanded fragments. In the case that more than one expanded fragment meets the criteria, we select the fragment with the most matches in the database. The process terminates when there are no remaining contacting residues to add, or none of the expanded fragments meet the criteria. The fragments that result from this protocol can be either single- or multi-segment and we refer to them as *complex* fragments. When defining complex fragments for this work, we used m_{max}= 2.

Generating seeds from structural matches to binding site fragments

Each protein structure containing a match to a binding site fragment provides a new structural context from which interface seeds can be generated. We use the alignment of the match to identify the residue in the match protein that corresponds to the central residue of the query, r_i^M. Then we identify the set of residues in the match protein that contact r_i^M (excluding those within the aligned region of the match): $r_j^M \in C(r_i^M)$. We define potential seed residues as the union of $C(r_i^M)$ and the m = 2 flanking residues on either side of r_j^M. The backbone atoms of the potential residues are placed in the space around the target protein by applying the transformation that optimally superimposes the match to the original query fragment. Potential seed residues with atoms that clash with the atoms of the target protein (backbone or sidechain) are considered non-designable and removed. Specifically, two heavy atoms a_i and a_j, one in the target protein and one in the potential seed residue, are considered clashing based on the following condition:

$$d_{i,j} \leq c\left(Rad_i + Rad_j\right) \tag{1}$$

Where $d_{i,j}$ is the Euclidian distance between a_i and a_j, Rad_i and Rad_j are the van der Waals radii (N = 1.6 Å, C = 2.1 Å, O = 1.6 Å) of atom a_i and a_j, respectively, and c is a constant in the range [0,1]. In this work we used c = 0.7, meaning that minor clashes were permitted. If a potential seed residue is found to clash with an atom from the protein, that residue is eliminated.

The remaining potential seed residues are grouped into segments by the connectivity of their peptide bonds, and each segment defines a distinct seed. In this work, if a seed had fewer than 5 residues, it was discarded. For each seed, we record the section of the match protein that aligns to the binding site fragment, which we refer to as the anchor. This section of the fragment is used later when joining seeds to design peptide backbones.

Finding pairwise seed alignments

We defined local alignments between seeds using geometric criteria. For two seeds s_A and s_B, we define a k-residue alignment starting at position i of s_A and j of s_B, if two criteria are satisfied. First, we consider a *distance condition* where the Euclidean distance between the corresponding C_α of each residue in the alignment can be no greater than the cutoff τ:

$$\max_{0 \le l < k} \left\| \boldsymbol{a}_{i+l} - \boldsymbol{a}_{j+l} \right\| \le \tau \tag{2}$$

where $\boldsymbol{a}_i$ and $\boldsymbol{a}_j$ are vectors describing the position of the C_α atom in residues r_i and r_j of seeds s_A and s_B, respectively. Second, a normal vector $\boldsymbol{r}_i$ is defined for each residue as the dot product of the N-C_α and C_α-C vectors. We define an *orientation condition* where the average angle between corresponding residue normal vectors $\boldsymbol{r}_{i+l}$ and $\boldsymbol{r}_{j+l}$ can be no less than θ_{max}:

$$\frac{1}{k} \sum_{0 \le l < k} \frac{\boldsymbol{r}_{i+l} \cdot \boldsymbol{r}_{j+l}}{\left\| \boldsymbol{r}_{i+l} \right\| \left\| \boldsymbol{r}_{j+l} \right\|} \ge \cos \theta_{max} \tag{3}$$

In this work we used k = 4 residues, τ = 1.0 Å, and θ_{max} = 45°.

The search for all k-residue alignments between all pairs of seeds is quadratic in both the size of the seed set and the length of the seeds. Because our seed sets contain thousands to millions of seeds, we developed a more efficient algorithm to find alignments by geometric hashing. First, we define a bounding box around the seeds and hash each seed residue by its three-dimensional C_α coordinate, binned at 0.3 Å intervals. This allows us to find the set of hashed seed residues with C_α within τ of some query residue, $T(r_i)$, in constant time. To find all alignments to a k-residue window of s_A starting at position i, we first generate sets $T(r_m)$ for $i \le m < i + k$. Each set of residues is then sorted by a function that considers the seed from which a residue originates and the position of the residue in the seed chain. After sorting, the order of residues from different seeds is arbitrary, but deterministic, and the order of the residues within the same seed is from N-to-C. The k sorted lists of seed residues are traversed using an operation similar to the combine step of the merge sort algorithm. This allows us to

quickly identify the case where each list contains a residue from another seed, s_B, in the proper order (e.g., $r_j, r_{j+1}, \ldots, r_{j+k-1}$), indicating alignment to s_A. The time complexity of this algorithm is linear in the number of seeds, and in practice completes in minutes to a few hours. After obtaining a set of candidate alignments and verifying that they satisfy the distance condition, we apply the orientation condition to obtain the final set of alignments.

Representing seeds and their pairwise alignments as a directed graph

We represent the seeds as a directed graph: $G = (V, E)$, where $r_i \in V$ is a residue in a seed, and $(r_i, r_{i+1}) \in E$ is a peptide bond between the C-terminal atom of r_i and the N-terminal atom of r_{i+1}. To build G, we first incorporate all of the seed residues and intra-seed peptide bonds. Next, we include *potential* peptide bonds, indicated by the pairwise seed alignments. Consider s_A and s_B, with a k-residue alignment starting at positions (i, j) in s_A and s_B, respectively. Given this alignment, we define directed edges between residues of the two seeds: $(r_{i+\frac{k}{2}-1}, r_{j+\frac{k}{2}})$ and $(r_{j+\frac{k}{2}-1}, r_{i+\frac{k}{2}})$. Note that k is chosen to be even (in this work, k = 4), and the directed edges are drawn between the residues at the center of the alignment: the most conservative joining point. Given this formulation, we can define a directed path $(r_1, r_2, \ldots, r_n)$, i.e., a sequence of seed residues connected by directed edges that can span distinct seeds. The residues in a directed path can be joined to generate a backbone using our algorithm, Fuser (see below).

We developed a strategy for sampling random directed paths from G. First, a single seed s_a is randomly selected from V, with a probability given by the number of residues in the seed. All residues in s_a are used to define the initial path: $(r_{a,1}, r_{a,2}, \ldots, r_{a,n})$. We attempt to extend the path using a depth-first search in the N- and C-terminal directions. If the C-terminal residue in the current path, $r_{a,n}$, has any outgoing edges, one of them is selected at random. The new residue, $r_{b,1}$, and all the residues from the seed in the C-terminal direction, $(r_{b,1}, r_{b,2}, \ldots, r_{b,m})$ are added to the path: $(r_{a,1}, r_{a,2}, \ldots, r_{a,n}, r_{b,1}, r_{b,2}, \ldots, r_{b,m})$. If $r_{a,n}$ has no outgoing edges, it is removed from the path and the extension process is attempted again, until no more outgoing edges are found. To extend the path in the N-terminal direction, the same process is repeated, starting at $r_{a,1}$. In practice, this yields diverse paths consisting of residues from multiple seeds.

Fusing seeds to yield peptide backbone designs

We used Fuser to join aligned protein fragments into a single backbone (i.e., the fused structure). Briefly, Fuser begins with the construction of a topology that maps the aligned fragments to the target fused structure, assigning correspondences between atoms. After building the topology and initializing a set of coordinates for the fused structure, the coordinates of each atom are optimized using an objective function that minimizes the difference between the fused backbone and the aligned fragments while maintaining realistic stereochemistry. When applying Fuser to generate peptides out of seeds, we construct a topology for a directed path in the context of the binding site; the topology includes both the target protein binding site and the seeds to be fused, with their respective anchors. Consider a

target protein binding site with n residues and a directed path with m residues. We construct a topology t with $l = n + m$ positions. First, we add the backbone coordinates for each residue of the target protein to a position in the topology: $(t_0, t_1, \dots, t_{n-1})$. Next, for each residue in the directed path, r_i (where r_i is the ith residue of the path), we add r_{i-1}, r_i, r_{i+1} to positions $(t_{n+i-1}, t_{n+i}, t_{n+i+1})$, and the anchor residues in the range $(t_0, t_1, \dots, t_n)$, with correspondence dictated by the alignment. The addition of residues flanking r_i helps provide structural context during later stages of fusing. When r_i is a terminal residue, i.e., $i = 0$ or $i = m$, only one flanking residue is added.

We update the coordinates of non-fixed atoms $a \in (t_n, t_{n+1}, \dots, t_{n+m-1})$ by minimizing the Fuser loss:

$$F(s; A) = \sum_{f \in A} RMSD(s_f, f) + IC(s)$$

where s is the fused structure and A is the set of fragments mapped to the topology. We calculate the root-mean-square deviation (RMSD) overall all backbone atoms after optimally superimposing fragment f to s_f, the section of the fused structure to which f is mapped. The internal coordinates loss consists of the following terms:

$$IC(s; c, v) = c_l \sum_{(a_i, a_j) \in s} length\_penalty(a_i, a_j) + c_a \sum_{(a_i, a_j, a_k) \in s} angle\_penalty(a_i, a_j, a_k) + c_d \sum_{(a_i, a_j, a_k, a_l) \in s} dihedral\_penalty(a_i, a_j, a_k, a_l)$$

Where a is an atom in s, and each c is an empirically-derived weight; in this work we used c_l = 10, c_a = 0.02, and c_d = 0.001. The three loss terms are harmonic penalties applied to all bonds in s that deviate from the allowable values v observed in the aligned fragments. The bond-length penalty is calculated for all bonded atoms and the bond-angle penalty is calculated for all three-atom connected components. The dihedral angle penalty is calculated for (N, C_α, C, O) at each position in s and for $(N_{-1}, C_{\alpha,-1}, C_{-1}, N)$, $(C_{\alpha,-1}, C_{-1}, N, C_\alpha)$, and $(O_{-1}, C_{-1}, N, C_\alpha)$ at all positions in s where $0 < i \le l - 1$.

We used gradient descent to minimize the Fuser loss with respect to the position of each atom in the fused structure that was not fixed. In this work, we consider the structure of the target protein to be fixed, so we initialize the coordinates of atoms at positions $(t_0, t_1, \dots, t_{n-1})$ to those of the target protein and hold them constant. The rest of the positions, $(t_n, t_{n+1}, \dots, t_{n+m-1})$, are initialized by averaging the coordinates of the atoms mapped to that position. Prior to each iteration of gradient descent, we optimally or re-superimposed each fragment f to the aligned section of the current fused structure s_f. We used an initial step size of 0.001 and allowed this to adaptively update depending on the magnitude of the gradient. Optimization halted after either 100 iterations or if the difference in RMSD between the

previous and current iteration of s was less than 10^{-4} Å. The source code is available in the following public repository: https://github.com/Grigoryanlab/Mosaist.

Scoring and ranking the designed peptide backbones

We developed the TERM interface score to quantify the compatibility between the structure of a peptide backbone and a target protein binding site. We identified contacts between residues of the peptide $r_i \in P$ and the target protein $t_j \in T$. For each interface contact (r_i, t_j), we defined a fragment from the contacting residues plus the m = 1 flanking residues on each side of each residue, which we denote the interface fragment f_i. We also defined a fragment containing only t_j and the m = 1 flanking residues on each side, which we denote the surface fragment f_s. We used FASST (see below) to search f_i and f_s against a database of biounit protein structures to yield M_i and M_s, the set of structural matches to each interface or surface fragment, respectively. The sequence statistics from the structural matches were used to compute the interface score:

$$Score(f_i\,, f_s) = -\ln\frac{P(a_n|f_i\,)}{P(a_n|f_s)}$$

$$P(a_n|f_i) = \frac{1}{|M_i|}\sum_{m\in M_i}[a_n = a_m]$$

$$P(a_n|f_s) = \frac{1}{|M_s|}\sum_{m\in M_s}[a_n = a_m]$$

(4)

Where $P(a_n|f_i)$ and $P(a_n|f_s)$ are the conditional probabilities of the native amino acid a_n at position t_j. The vales are estimated by computing the fraction of structural matches $m \in M$ where the amino acid a_m (at the position in the match that corresponds to t_j) has the native identity. This score is negative when the probability of the amino acid on the protein target is higher in the structural matches to the interface fragment vs. the surface fragment alone. The score is computed per interface contact and averaged over all contacts to get the overall score for the peptide-protein interface.

Designing sequences on peptide backbones with dTERMen

We used dTERMen to design amino-acid sequences for candidate peptide backbones. dTERMen extracts sequence-structure relationships from a database of known structures and can be used to construct a Potts model (e.g., a collection of self and pair energy parameters) that captures the probability of any sequence given a structure[25]. We modified the dTERMen procedure by limiting the size of cliques used to estimate the near-backbone self-correction factor (maximum clique size = 2), which offered a considerable improvement in runtime. We obtained TERM statistics by searching BioUnitDB and filtering out all structural matches with sequence homology (see below).

We designed optimal sequences for peptide backbones using Markov chain Monte Carlo Simulated Annealing (MCMC SA) with the dTERMen Potts model. At each step a random mutation was proposed, from the set of 20 native amino acids, and then accepted or rejected according to the Metropolis criterion. We initialized the search with a random sequence and performed 100,000 iterations. This was repeated 100 times and the lowest sequence encountered was selected as the optimal sequence. Altogether, this process takes approximately 1 second. The annealing schedule was linear, starting at kT = 1.0 and terminating at kT = 0.01. The lowest energy sequence encountered was selected as the optimal sequence. The design was performed in the context of the target protein structure and sequence, which were fixed.

Searching for structural matches

We use the program FASST to search for matches to structural fragments in protein structure databases. The FASST algorithm guarantees that all structural matches to the query within a pre-determined RMSD cutoff, R_{cut}, are identified. A match consists of a set of aligned backbone atoms (i.e., N, C_α, C, O) within a protein in the database with $RMSD(query, match) \leq R_{cut}$ after optimal superposition. The underlying theory of the search algorithm and RMSD bounds are described in Zhou et. al[53]. The source code for the current version of FASST is available at: https://github.com/Grigoryanlab/Mosaist.

The exact R_{cut} that is most appropriate for establishing the structure match of a query is difficult to define, because RMSD depends on the size and complexity of the query. Following previous work, we used a size and topology dependent function with default parameters to define R_{cut}[24]. In this work, we used R_{max}=1.2 Å. R_{cut} only approached 1.2 Å when searching the largest fragments; the majority of the fragments consisted of a single segment and a stringent RMSD cutoff of <= 0.5 Å was sufficient to identify many matches.

In all applications of FASST in this work, we removed structural matches that were considered homologous by sequence identity. We compared each match to the query using the aligned residues and a sequence window including up to 30 N- and C- terminal residues flanking the aligned region. Matches with sequence identity to the target of 50% or greater over this window were removed.

Assembling non-redundant structure databases to search with FASST

We constructed two FASST structure databases from a non-redundant, high-quality subset of structures from the PDB. We downloaded all biological assembly files from the PDB on January 22, 2019. We removed all membrane proteins, structures obtained by NMR, and X-ray crystallography structures with resolution worse than 2.6 Å. We also filtered out structures with more than 5000 residues, structures with more than 26 chains, and structures with more than 40% non-protein atoms.

The first database consisted of single-chain structures (SingleChainDB) and the second database consisted of biological units, some of which consisted of multiple chains (BioUnitDB).

To generate the single-chain database, we split up structures with more than one chain such that there was only one chain per structure and we removed any structure with fewer than 25 residues. To eliminate redundancy due to sequence homology, we used uClust[54] to cluster the remaining structures by sequence identity, with a threshold of 30%. We then selected the highest resolution structure from each cluster, resulting in 22,183 structures and 5,449,800 residues (see Table 6). To generate the biological units database, we left all structures in their biological assembly, resulting in a mixture of single- and multi-chain structures. We used uClust to cluster all chains of the structures with a sequence identity threshold of 50%. A structure was considered redundant to another if each of its chains was redundant (i.e., fell in the same cluster) to a chain in the other structure. If one structure had fewer chains than another it was redundant to, it was eliminated. In cases where two redundant structures had the same total number of chains, the structure with lower resolution was eliminated. The final biological units database contained 23,580 structures, with 70,622 chains total, and 10,225,565 residues (see Table 7).

Generating decoy seeds

For each set of TERM seeds, we generated a corresponding set of decoy seeds that shared most properties of the TERM seeds but had random orientation and conformation. We replaced each TERM seed with a random, equal-length segment of backbone randomly sampled from the structural database. This ensured that the number of TERM seeds and their lengths were preserved in the decoy set. Next, we sampled a random position and orientation for each seed and applied a rejection criterion to ensure that distance from the protein distribution of the decoy seeds matched that of the TERM seeds. Positions were sampled uniformly within a bounding box encapsulating the peptide with 10 Å of padding. If the seed clashed with the protein, a new conformation and pose were sampled. Once a viable seed was placed, we found the distance, *d*, between the seed centroid and the closest protein backbone atom and applied rejection sampling with the following acceptance condition:

$$U \leq \frac{f(d)}{m * g(d)} \tag{5}$$

U is a randomly sampled value from the uniform distribution $[0,1]$. $f(d)$ and $g(d)$ are the proposal and target probability density functions, respectively. m is a constant with a value such that $m * g(d) \geq f(d)$ for all values of d, which ensures that the target distribution envelopes the proposal distribution. We approximate the probability density functions as normalized histograms, which are described below. When a seed was rejected, a new conformation and pose were sampled, for that same seed, and rejection sampling was applied again until it was accepted.

A proposal distance distribution for each set of TERM seeds was made from a corresponding set of random seeds without rejection sampling. Distances were computed

between each seed and the protein, binned using 0.25 Å intervals, and normalized. The target distance distribution was generated using TERM seeds from 9 complexes (list in the supplement). A normalized histogram was generated for each set of seeds and these were averaged to give a generalized target distribution. This procedure ensured that for each seed in the TERM set, there was a seed of equal length in the decoy set, that none of these seeds clashed with the protein, and that the overall distance distribution of decoy seeds matched that of the target distribution.

Supplementary Figures (S1-S22)

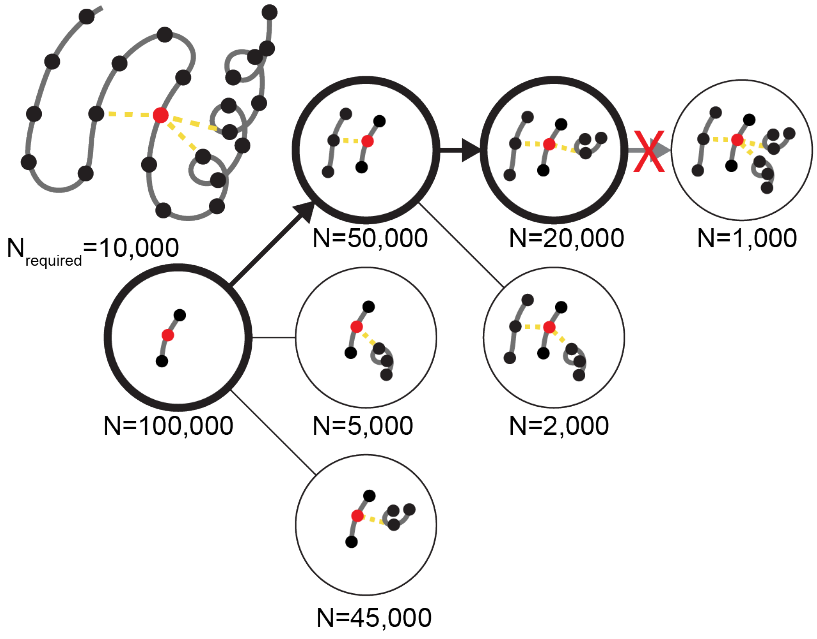

Figure S1. Example to illustrate the greedy algorithm for generating complex fragments, with m = 1, $N_{required}$ = 10,000. The central residue (red) and the contacting residues (contacts indicated with yellow dashed lines) are identified in the target protein. A single-segment fragment is defined around the central residue. Each contacting residue is added individually to create a separate expanded fragment. The number of matches to each fragment is shown. The fragment with the most residues, with at least N matches in the structural database, is selected. This fragment is expanded again by adding each of the remaining contacts, but after this round the process terminates, as there are insufficient matches.

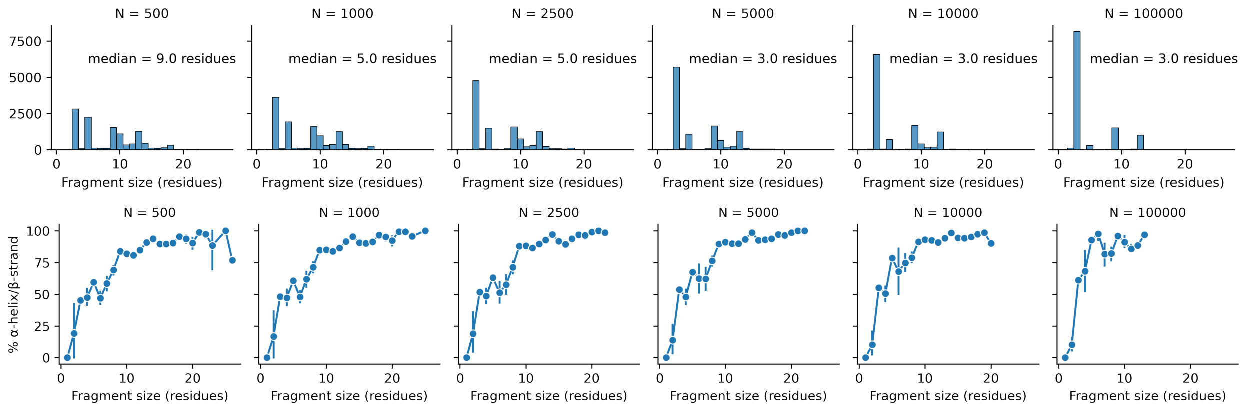

Figure S2. Distribution of fragment sizes (top) and the average percentage β-beta strand or helix composition of fragments (bottom) for different values of *N* when searching for matches in the SingleChainDB.

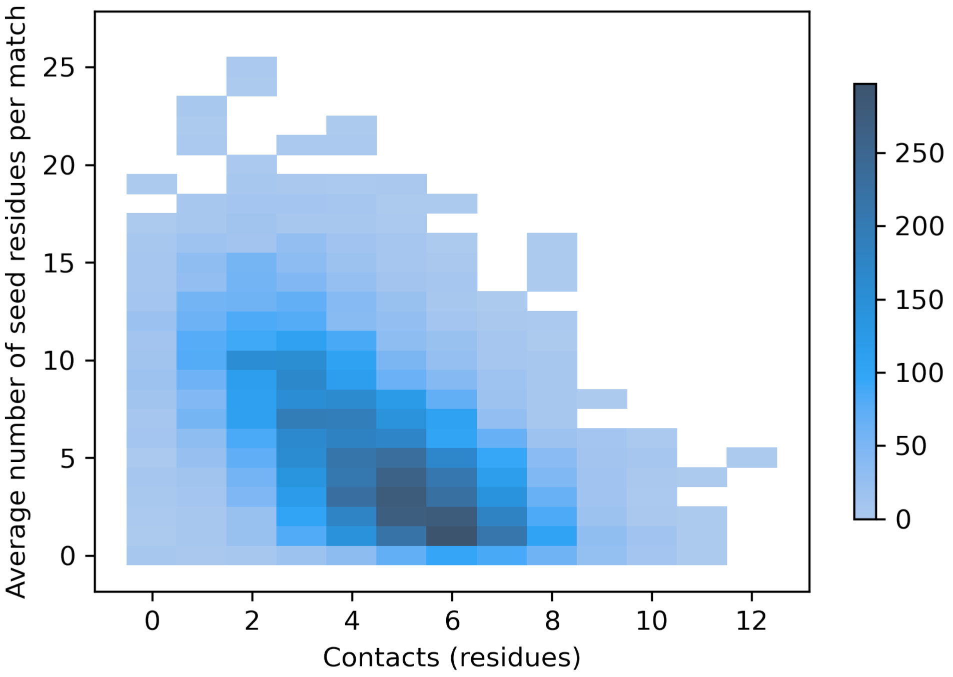

Figure S3. The average number of seed residues per match is negatively correlated with the number of protein contacts to the central residue used to define the fragment. Results shown for PixelDB-486, when *N* = 100,000 matches, and searching SingleChainDB. The number of fragments per bin is shown by the color scale to the right.

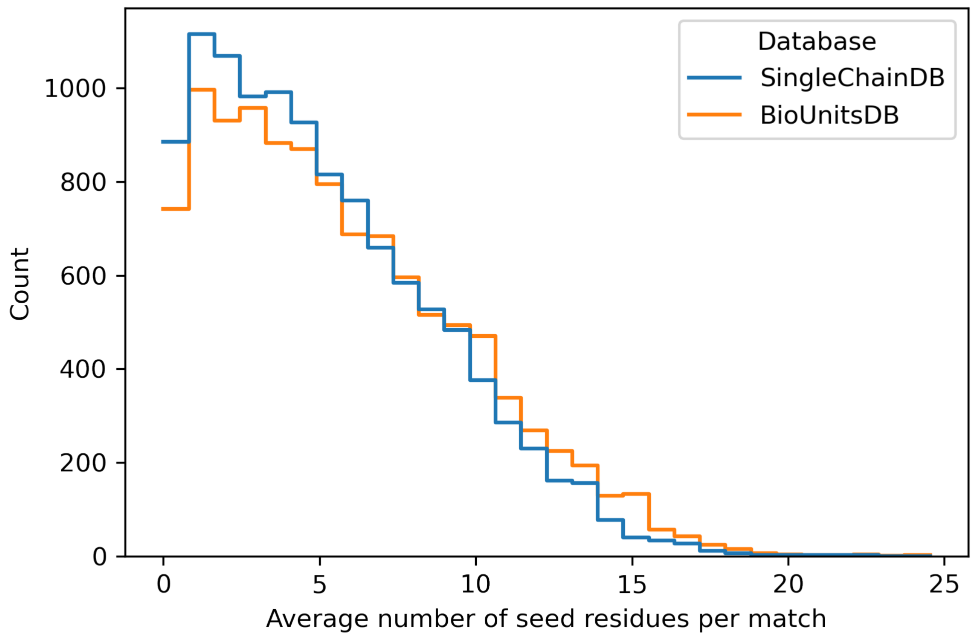

Figure S4. The distribution of the average number of seed residues per match is shown when fragments are generated from PixelDB-486 binding sites and when searching either the SingleChainDB or BioUnitsDB.

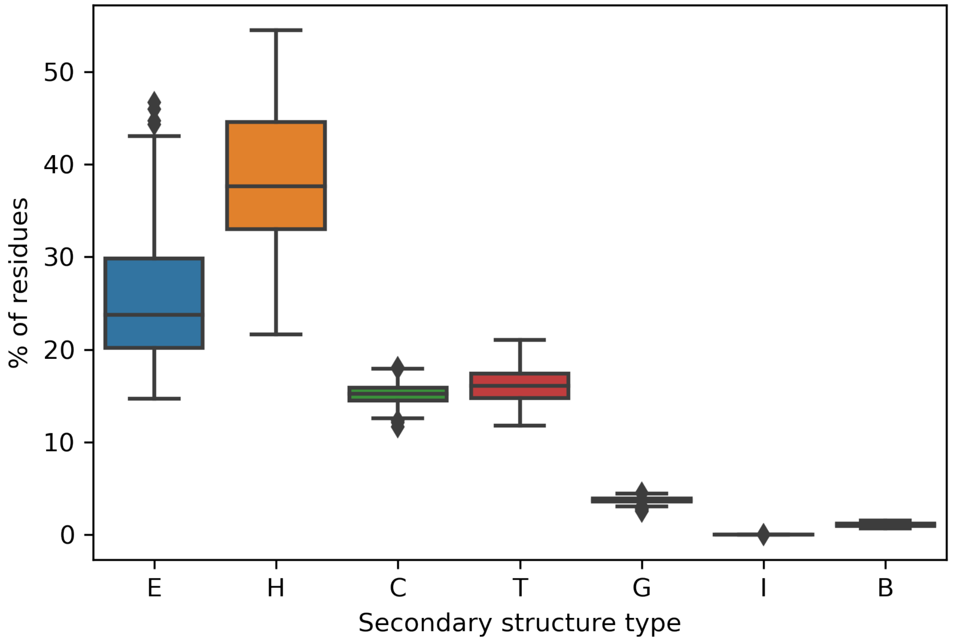

Figure S5. Secondary structure of seeds varies between binding sites. Seeds generated around PixelDB-486 binding sites with N = 100,000 matches. Labels: H = α-helix, E = β-strand, T = turn, C = coil, G = 3-10 helix, B = bridge, I = pi-helix.

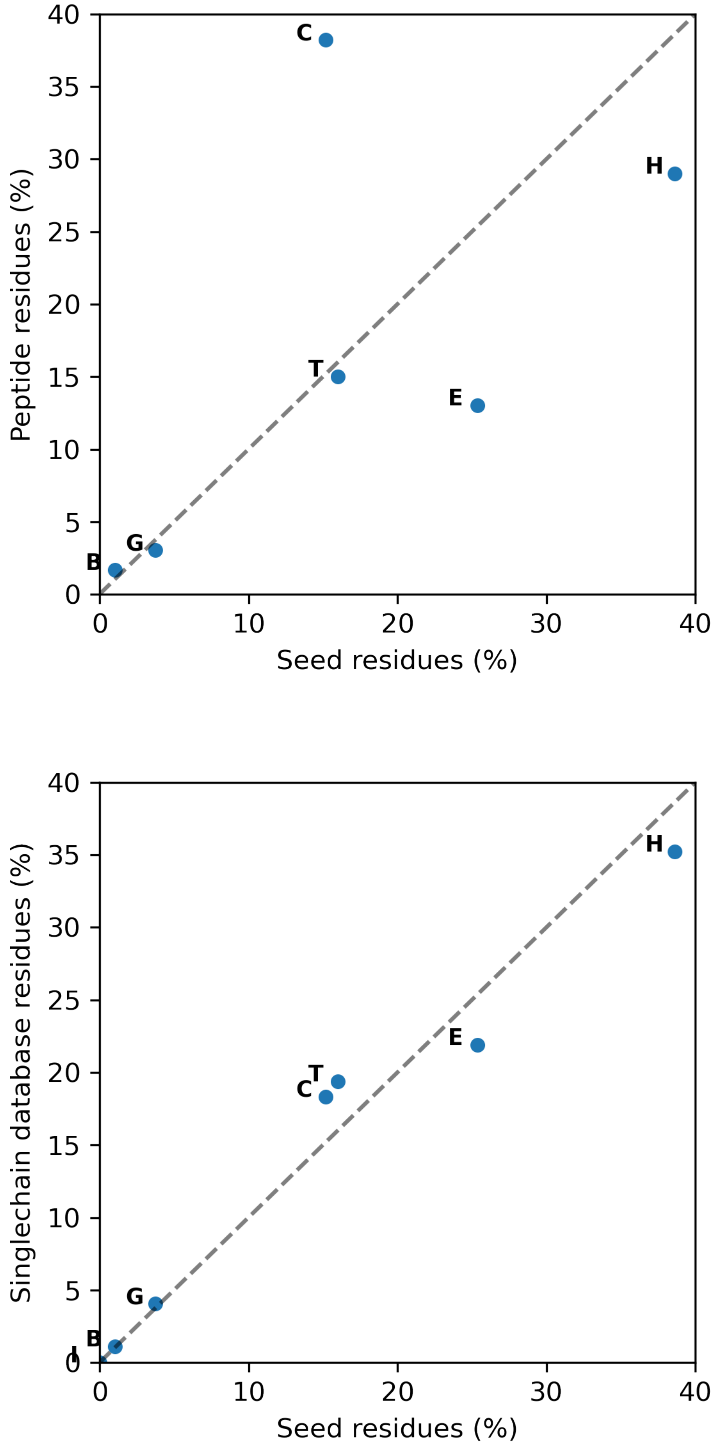

Figure S6. The overall secondary structure composition of seed residues generated around all binding sites in PixelDB-486 (SingleChainDB, *N* = 100,000) is compared to PixelDB-486 peptide residues (top) and SingleChainDB (bottom). Secondary structure labels are as in Figure S5.

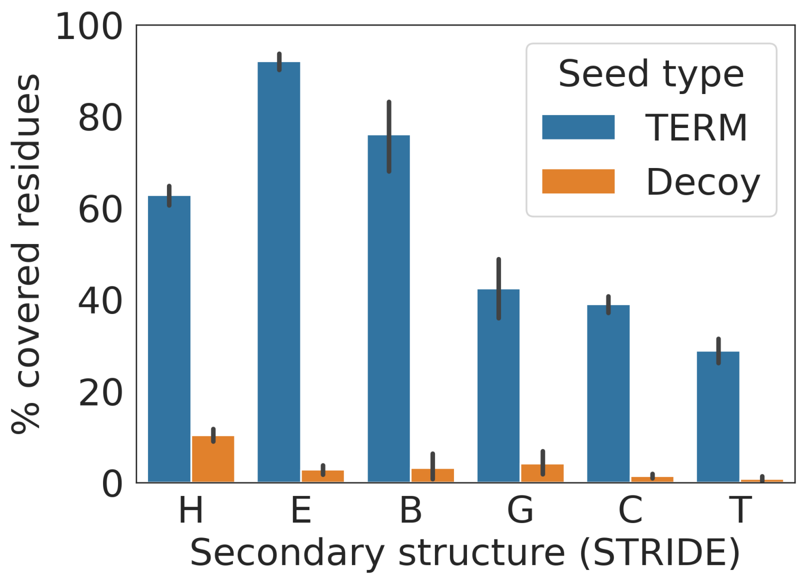

Figure S7. Coverage by peptide residue secondary structure when the top 2,500 matches are used to generate seeds. Secondary structure labels are as in Figure S5.

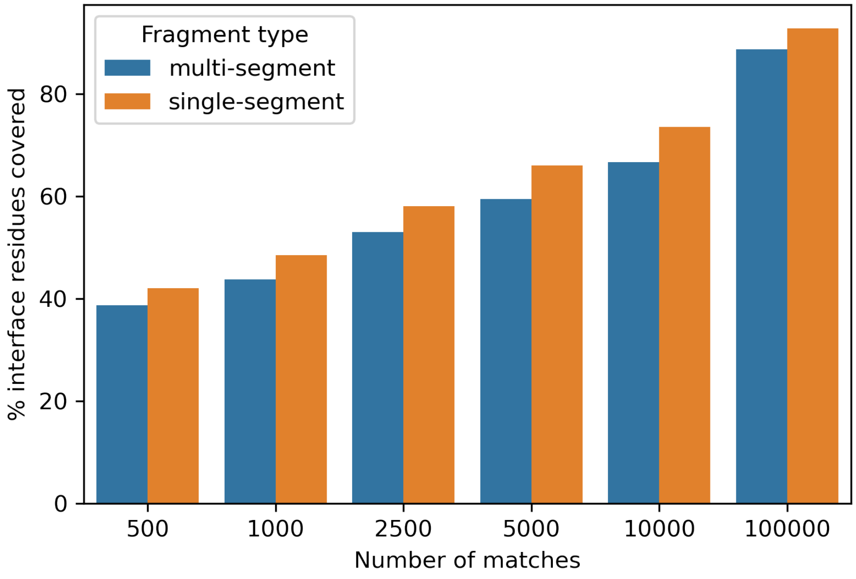

Figure S8. The percentage of peptide interface residues that are covered when generating seeds from single-segment vs. multi-segment (i.e., complex) fragments with equivalent numbers of matches.

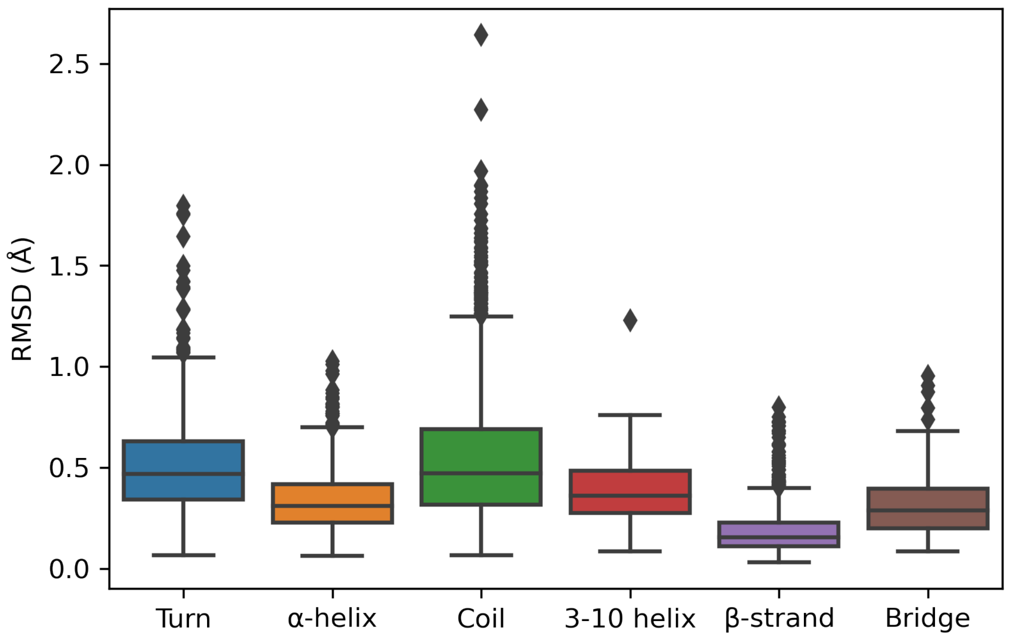

Figure S9. The per-residue RMSD between reconstructed and native residues by secondary structure of the native peptide.

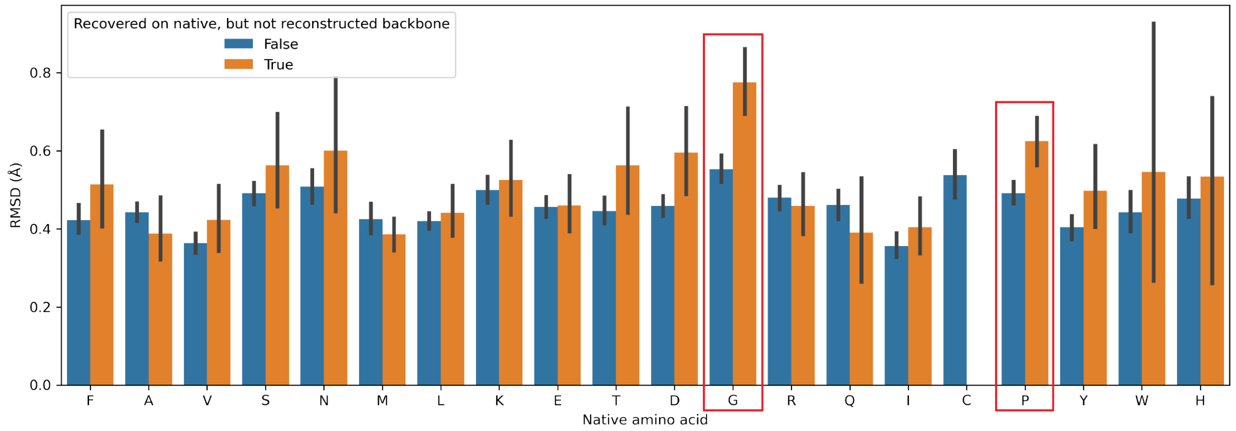

Figure S10. The RMSD of reconstructed to native residues, grouped by native amino acid. The values are further grouped into A) positions where the amino acid is recovered on the native, but not reconstructed backbone (orange) and B) all other positions. The red boxes indicate native amino acids where the RMSD was significantly different between the two groups.

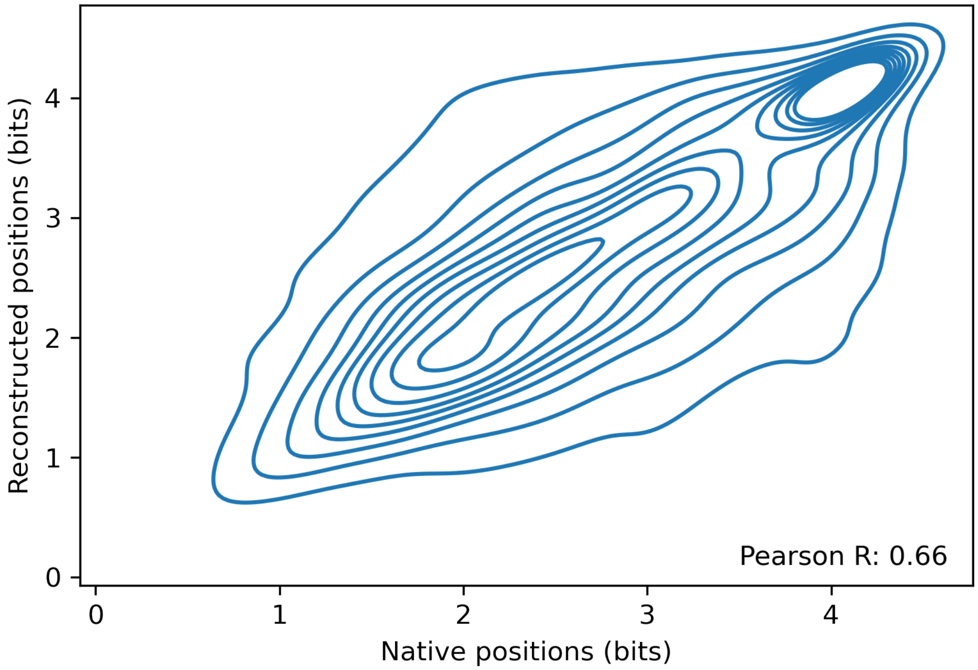

Figure S11. Kernel density estimate of the sequence conservation per position of native vs. reconstructed peptides when sampling sequences with MCMC SA.

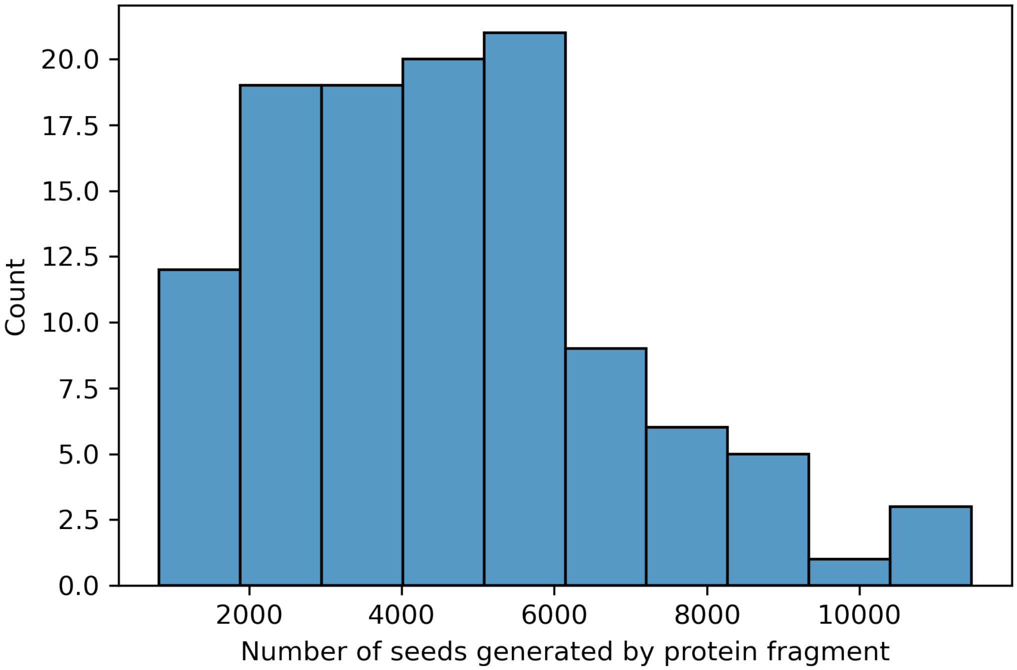

Figure S12. The distribution of number of seeds generated from surface fragments (i.e., fragments defined around a central residue with relative SASA >= 0.05).

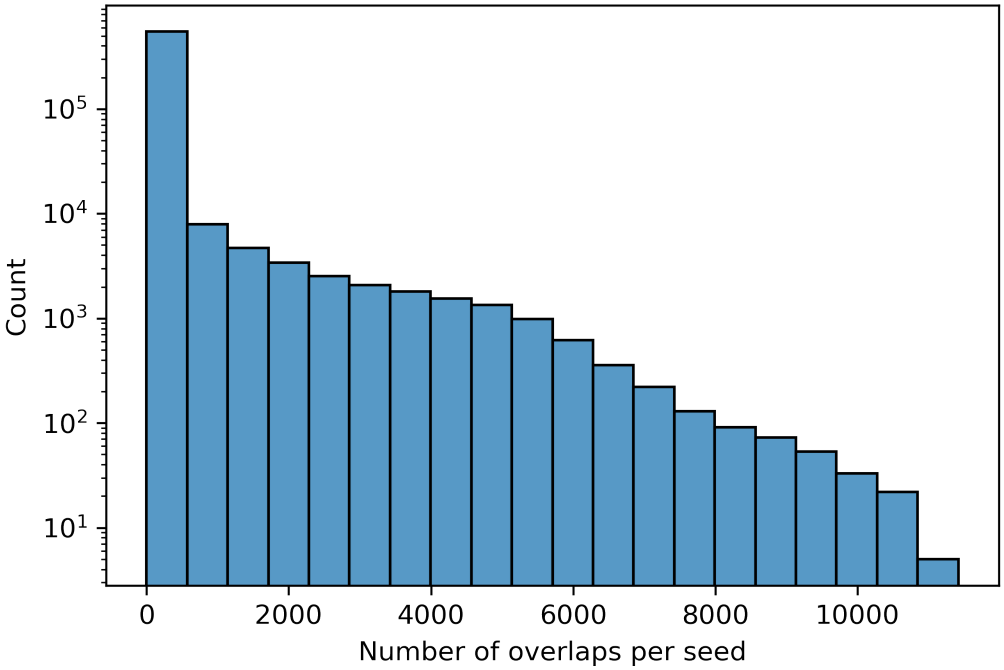

Figure S13. The distribution of four-residue overlaps per seed.

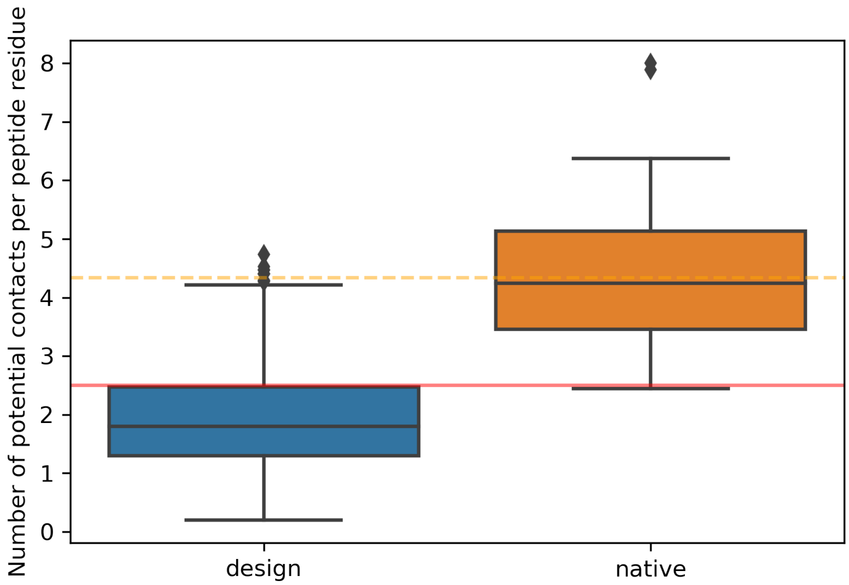

Figure S14. Number of potential contacts per designed peptide or native peptides from PixelDB-30. The red line indicates the cutoff used to filter designs. The orange dashed line corresponds to the value for native peptide CD40.

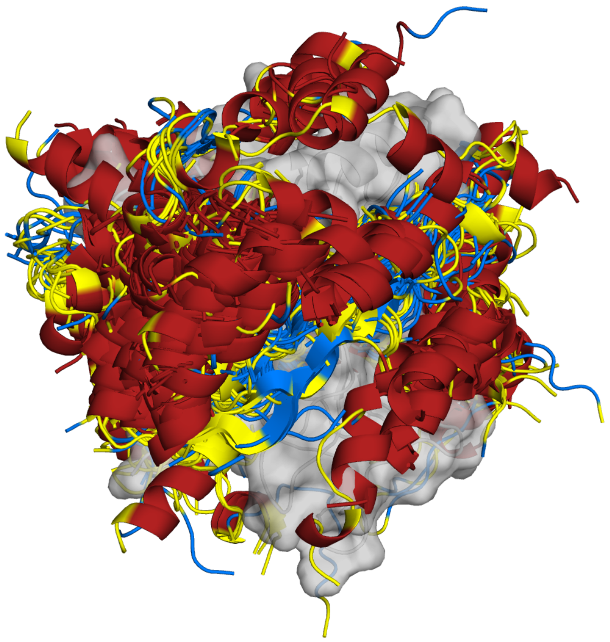

Figure S15. 986 designed peptide backbones with more than 2.5 contacts per residue. Secondary structure colors assigned as in Figure 4.

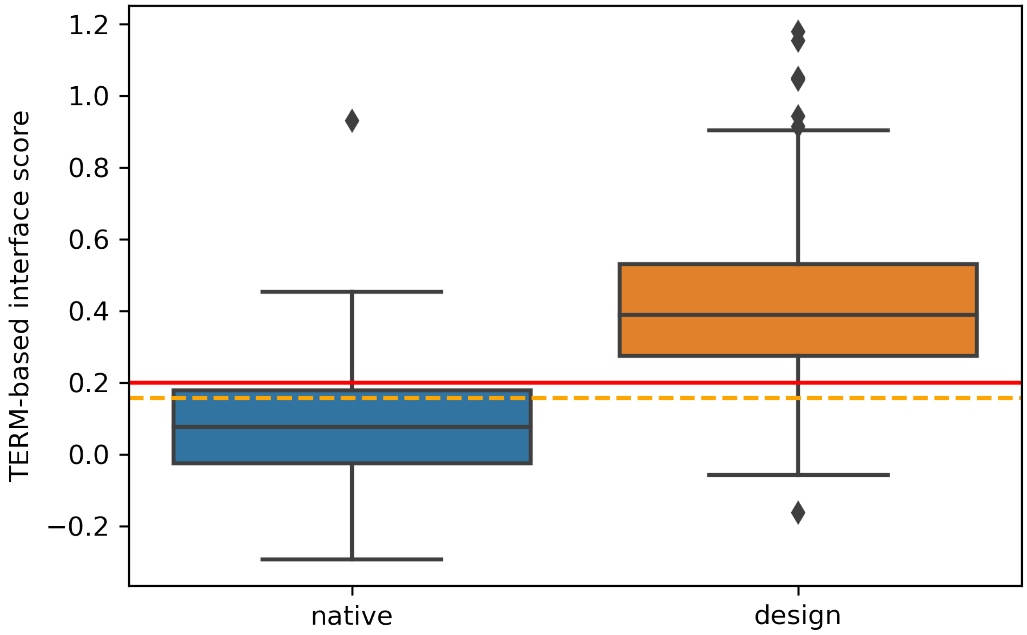

Figure S16. Average TERM-based interface score per designed peptide or native peptides from PixelDB-30. The red line indicates the cutoff used to filter designs. The orange dashed line corresponds to the native binder CD40.

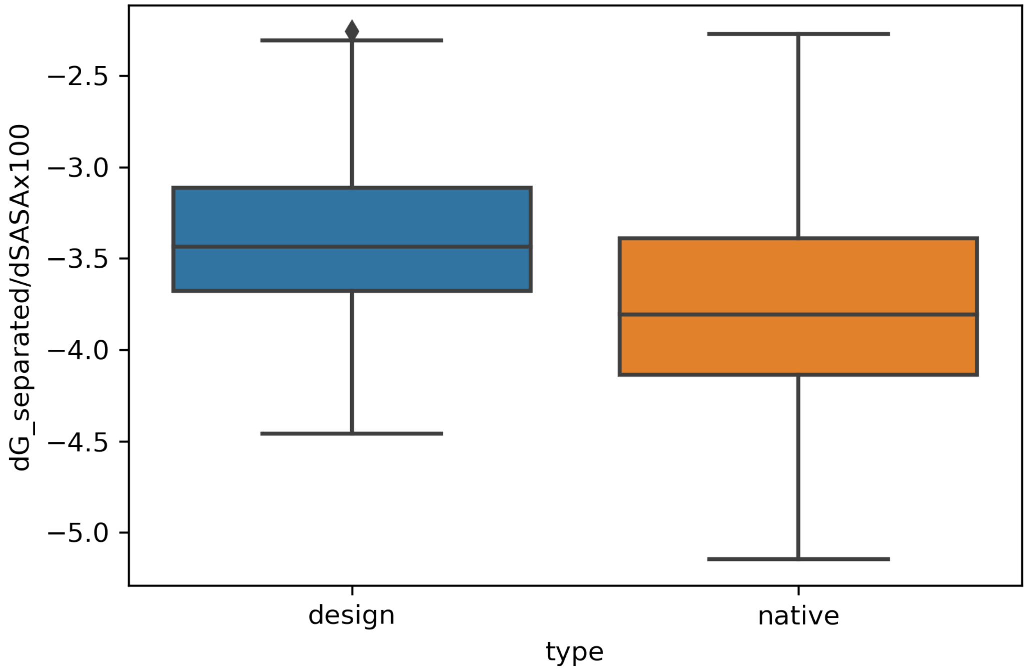

Figure S17. ΔGbind/ΔSASA (Rosetta interface score) for the designs vs. native peptides.

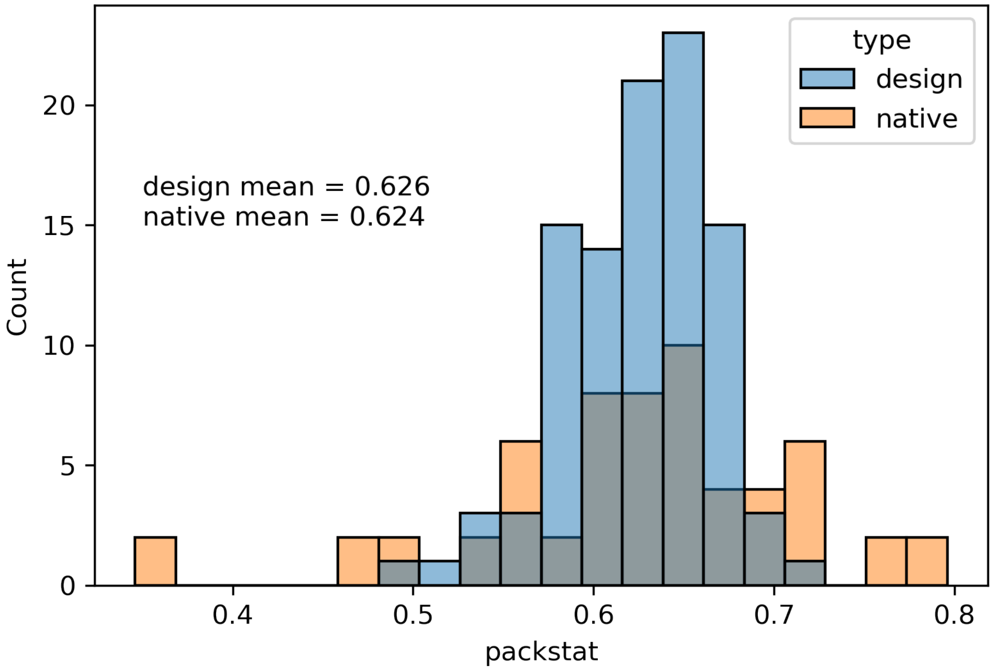

Figure S18. Packstat computed for 100 final designs and native peptides from PixelDB-30 after Rosetta relax.

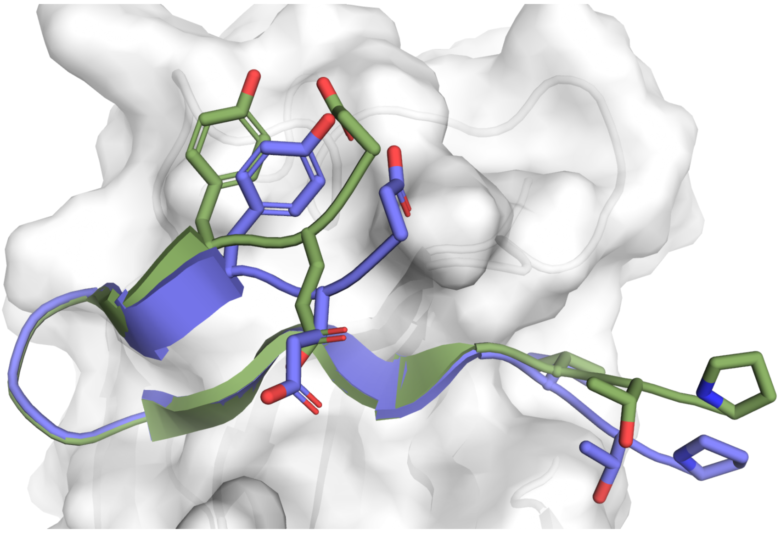

Figure S19. The minimum energy structure (purple) of peptide A2 from FPD Refine deviates from the Rosetta Relaxed structural model (green) at the termini.

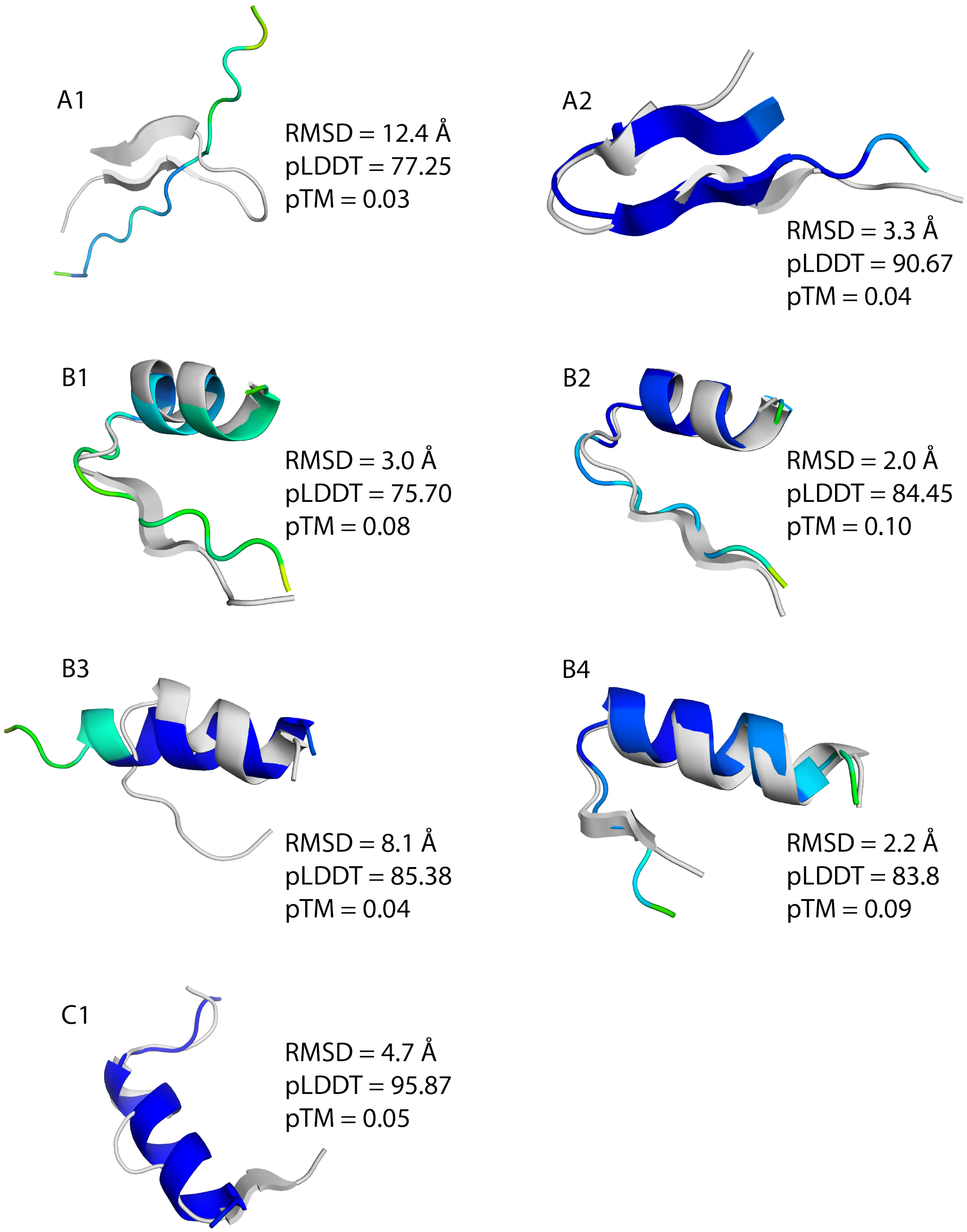

Figure S20. ColabFold peptide structure predictions. For each of the peptides, the top-ranked predicted structure (highest mean pLDDT) is shown superimposed with the Rosetta relaxed designed structure. The reported RMSD is over all heavy atoms, including sidechains. The AlphaFold model is shown colored by pLDDT (red/yellow: ~70, blue: 100), the Rosetta-relaxed structures are shown in white.

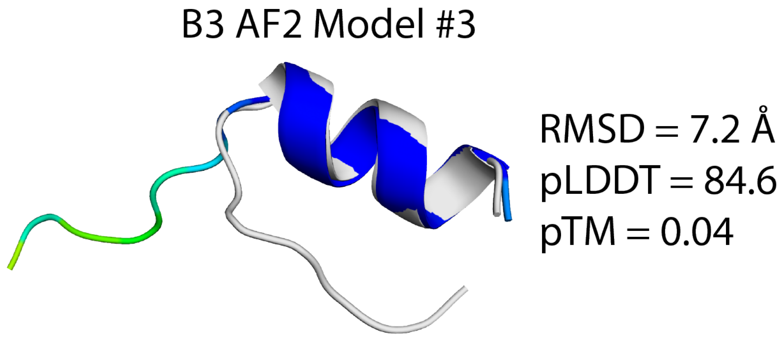

Figure S21. ColabFold third-ranked structure prediction for B3.

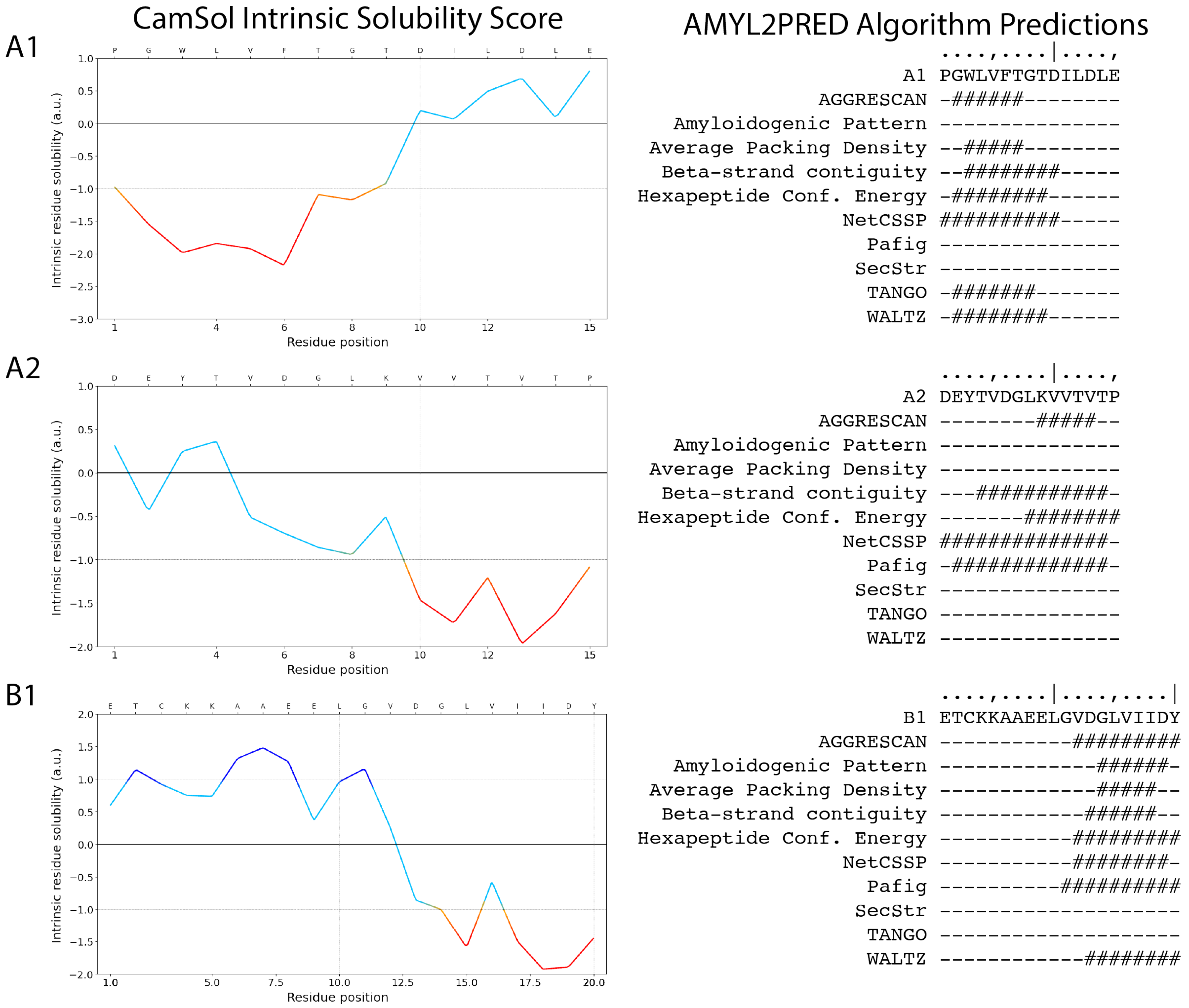

Figure S22. CamSol intrinsic solubility scores and AMYL2PRED individual residue scores for peptides predicted to aggregate. In the CamSol plots, red indicates poorly soluble, whereas dark blue indicates highly soluble. In the AMYL2PRED results, a hash indicates that the position is predicted by one of the algorithms to increase the potential for amyloid aggregation.

Supplementary tables (S1-S3)

Path type	Total	Path is a single seed with no overlaps	Less than 15 residues	Identical to a previously accepted path	Contains a VDW clash with the protein	Meets all criteria, accepted
**Number sampled**	154,847	88,176	61,933	461	277	4,000

Table S1. TRAF6 design path sampling statistics.

Design Name	Sequence
A1	PGWLVFTGTDILDLE
A2	DEYTVDGLKVVTVTP
B1	ETCKKAAEELGVDGLVIIDY
B2	EEFIEELKKAGFKNIAVGED
B3	GELARELRAAGHEVGGD
B4	DEFGEDLAEELKKAGHKVGG
C1	EALSEEQRKGLKELIG

Table S2. Top-ranked designed peptide sequences

Number charged residues	Sequence	dTERMen score	CamSol solubility score	AMYL2PRED aggregation score	Rosetta interface score	Interface SASA (Å^2)	Interface hydrogen bonds
4	DEYTVDGLKVVTVTP	10.7629	0.811425	5	-4.356	1196.009	7
5	DEVEVDGLRFVTVTP	11.0277	0.963741	6	-4.157	1213.064	5
6	EEYTVDGLKVVEVDP	11.1161	1.717128	3	-4.091	1214.375	6
7	EEYEVDGLRFVEVDP	11.4436	1.912745	4	-3.811	1310.154	6
8	KEEEVDGLRFVEVDP	12.0545	2.451082	3	-4.037	1265.564	6
9	KEEEVDGKRFVEVDP	13.285	2.643799	1	-3.286	1292.324	5
10	KEEEVDGKRFVEVDD	14.7459	2.687977	1	-3.751	1234.309	6
11	EEKEIDGKREVEVDD	17.4211	2.903727	1	-3.664	1413.164	8

Table S3. A2 sequence after redesign with a charge constraint and relevant statistics
